# The Oxygen Paradox, the French Paradox, and age-related diseases

**DOI:** 10.1007/s11357-017-0002-y

**Published:** 2017-12-21

**Authors:** Joanna M. S. Davies, Josiane Cillard, Bertrand Friguet, Enrique Cadenas, Jean Cadet, Rachael Cayce, Andrew Fishmann, David Liao, Anne-Laure Bulteau, Frédéric Derbré, Amélie Rébillard, Steven Burstein, Etienne Hirsch, Robert A. Kloner, Michael Jakowec, Giselle Petzinger, Delphine Sauce, Florian Sennlaub, Isabelle Limon, Fulvio Ursini, Matilde Maiorino, Christina Economides, Christian J. Pike, Pinchas Cohen, Anne Negre Salvayre, Matthew R. Halliday, Adam J. Lundquist, Nicolaus A. Jakowec, Fatima Mechta-Grigoriou, Mathias Mericskay, Jean Mariani, Zhenlin Li, David Huang, Ellsworth Grant, Henry J. Forman, Caleb E. Finch, Patrick Y. Sun, Laura C. D. Pomatto, Onnik Agbulut, David Warburton, Christian Neri, Mustapha Rouis, Pierre Cillard, Jacqueline Capeau, Jean Rosenbaum, Kelvin J. A. Davies

**Affiliations:** 1 The Medical Group, Internal Medicine, Rheumatology & Osteoporosis, Dermatology, Pulmonology, Ophthalmology, and Cardiology; the Hospital of the Good Samaritan, Los Angeles, CA 90017 USA; 2 Leonard Davis School of Gerontology of the Ethel Percy Andrus Gerontology Center, University of Southern California, Los Angeles, CA 90089-0191 USA; 3 Lab de Biologie Cellulaire et Végétale, Faculté de Pharmacie, Université de Rennes, 35043 Rennes Cedex, France; 4 Institut de Biologie Paris-Seine (IBPS), UMR CNRS 8256, Biological Adaptation and Ageing, Sorbonne Universités, UPMC Univ Paris 06, 75005 Paris, France; 5 INSERM ERL U1164, 75005 Paris, France; 6 School of Pharmacy, University of Southern California, Los Angeles, CA 90089-9121 USA; 7 Department of Biochemistry & Molecular Medicine, Keck School of Medicine of USC, University of Southern California, Los Angeles, CA 90033 USA; 8 Département de Médecine nucléaire et Radiobiologie, Faculté de médecine et des sciences de la santé, Université de Sherbrooke, Sherbrooke, Québec J1H 5N4 Canada; 9 Institut de Génomique Fonctionnelle de Lyon,ENS de Lyon, CNRS, 69364 Lyon Cedex 07, France; 10 Laboratory for Movement, Sport and Health Sciences-EA 1274, M2S, Université de Rennes 2-ENS, Bruz, 35170 Rennes, France; 11 INSERM UMR 1127-CNRS UMR 7225, Institut du cerveau et de la moelle épinière-ICM Thérapeutique Expérimentale de la Maladie de Parkinson, Université Pierre et Marie Curie, 75651 Paris Cedex 13, France; 12 Huntington Medical Research Institutes, Pasadena, CA 91105 USA; 13 Department of Neurology, Keck School of Medicine, University of Southern California, Los Angeles, CA 90033 USA; 14 Chronic infections and Immune ageing, INSERM U1135, Hopital Pitie-Salpetriere, Pierre et Marie Curie University, 75013 Paris, France; 15 INSERM Equipe 14 Institut de la Vision, 75012 Paris, France; 16 Department of Molecular Medicine, University of Padova, 35121 Padova, Italy; 17 Los Angeles Cardiology Associates, Hospital of the Good Samaritan, Los Angeles, CA 90017 USA; 18 Division of Neurobiology, Department of Biological Sciences of the Dornsife College of Letters, Arts, and Sciences, University of Southern California, Los Angeles, CA 90089-0191 USA; 19 Keck School of Medicine of USC, University of Southern California, Los Angeles, CA 90033 USA; 20 Lipid peroxidation, Signalling and Vascular Diseases INSERM U1048, 31432 Toulouse Cedex 4, France; 21 Stress and Cancer Laboratory, Institut Curie-Inserm U830, 75248 Paris Cedex 05, France; 22 Laboratoire de Signalisation et Physiopathologie Cardiovasculaire-Inserm UMR-S 1180, Faculté de Pharmacie, Université Paris-Sud, 92296 Châtenay-Malabry, Paris, France; 23 Department of Radiation Oncology, Hospital of the Good Samaritan, Los Angeles, CA 90017 USA; 24 Department of Oncology & Hematology, Hospital of the Good Samaritan, Los Angeles, CA 90017 USA; 25 Division of Molecular & Computational Biology, Department of Biological Sciences of the Dornsife College of Letters, Arts, and Sciences, University of Southern California, Los Angeles, CA 90089-0191 USA; 26 Children’s Hospital of Los Angeles, Developmental Biology, Regenerative Medicine and Stem Cell Therapeutics program and the Center for Environmental Impact on Global Health Across the Lifespan at The Saban Research Institute, Los Angeles, CA 90027 USA; 27 Department of Pediatrics, Keck School of Medicine of USC, University of Southern California, Los Angeles, CA 90033 USA; 28 DR Saint-Antoine UMR_S938, UPMC, Inserm Faculté de Médecine, Université Pierre et Marie Curie, 75012 Paris, France; 29 Scientific Service of the Embassy of France in the USA, Consulate General of France in Los Angeles, Los Angeles, CA 90025 USA

**Keywords:** Oxidative stress, Oxygen Paradox, Age-related diseases, French Paradox, Ageing, Proteostasis, Adaptive Homeostasis, Healthspan

## Abstract

A paradox is a seemingly absurd or impossible concept, proposition, or theory that is often difficult to understand or explain, sometimes apparently self-contradictory, and yet ultimately correct or true. How is it possible, for example, that oxygen “a toxic environmental poison” could be also indispensable for life (Beckman and Ames Physiol Rev 78(2):547–81, 1998; Stadtman and Berlett Chem Res Toxicol 10(5):485–94, 1997)?: the so-called Oxygen Paradox (Davies and Ursini 1995; Davies Biochem Soc Symp 61:1–31, 1995). How can French people apparently disregard the rule that high dietary intakes of cholesterol and saturated fats (e.g., cheese and paté) will result in an early death from cardiovascular diseases (Renaud and de Lorgeril Lancet 339(8808):1523–6, 1992; Catalgol et al. Front Pharmacol 3:141, 2012; Eisenberg et al. Nat Med 22(12):1428–1438, 2016)?: the so-called, French Paradox. Doubtless, the truth is not a duality and epistemological bias probably generates apparently self-contradictory conclusions. Perhaps nowhere in biology are there so many apparently contradictory views, and even experimental results, affecting human physiology and pathology as in the fields of free radicals and oxidative stress, antioxidants, foods and drinks, and dietary recommendations; this is particularly true when issues such as disease-susceptibility or avoidance, “healthspan,” “lifespan,” and ageing are involved. Consider, for example, the apparently paradoxical observation that treatment with low doses of a substance that is toxic at high concentrations may actually induce transient adaptations that protect against a subsequent exposure to the same (or similar) toxin. This particular paradox is now mechanistically explained as “Adaptive Homeostasis” (Davies Mol Asp Med 49:1–7, 2016; Pomatto et al. 2017a; Lomeli et al. Clin Sci (Lond) 131(21):2573–2599, 2017; Pomatto and Davies 2017)**;** the non-damaging process by which an apparent toxicant can activate biological signal transduction pathways to increase expression of protective genes, by mechanisms that are completely different from those by which the same agent induces toxicity at high concentrations. In this review, we explore the influences and effects of paradoxes such as the Oxygen Paradox and the French Paradox on the etiology, progression, and outcomes of many of the major human age-related diseases, as well as the basic biological phenomenon of ageing itself.

Outline

1. Basic Aspects of the Oxygen Paradox and Aging
2. The Role of Adaptive Homeostasis in the Oxygen Paradox
3. Heart and Circulatory System
4. Myocardial Ischemia and Reperfusion Injury
5. Lung Injury, Oxygen, and Acute Respiratory Distress Syndrome (ARDS)
6. Liver and The French Paradox
7. Cancer and carcinogenesis
8. Neurological Disorders and Stroke
9. Joints and Inflammation
10. Bone and Macrophages
11. Role of oxidation in Skin Photoaging
12. Cataracts
13. Age-related Macular Degeneration
14. Conclusions
15. Acknowledgements
16. References

## Basic aspects of the Oxygen Paradox, the French Paradox, and ageing

**Fig. 1:**
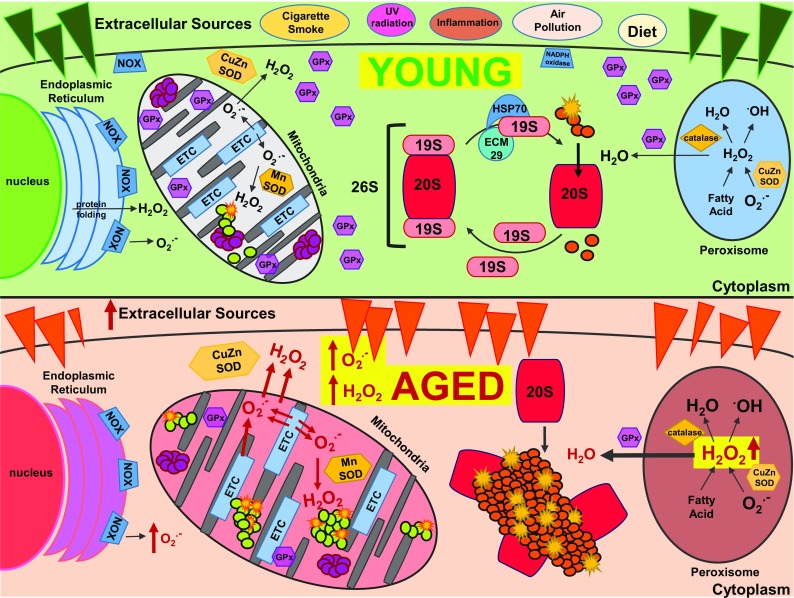
Sources of oxidative stress, cellular defenses, and compromised stress resistance with ageing. Upper (green background) panel—young cells—cells must cope with both internal and external sources of oxidative stress. Extracellular sources include smoke and partial combustion products (of all forms), pollution, radiation, environmental toxicants, and various dietary constituents. One internal source of oxidative stress arises from mitochondrial electron transport and respiration. Electron “leakage” from the electron transport chain to molecular oxygen generates superoxide (O_2_^·−^) which is either dismutated into hydrogen peroxide (H_2_O_2_) via the mitochondrial manganese superoxide (MnSOD) or the cytosolic copper-zinc superoxide (CuZnSOD). Additionally, if the superoxide is not removed and/or an accumulation of hydrogen peroxide (above homeostatic levels) occurs, the likelihood of hydroxyl radical (HO^·^) generation increases, with consequent protein, lipid, and even DNA damage. To combat these potential sources of damage, mitochondria rely upon detoxification enzymes, such as glutathione peroxidase (GPx) and proteolytic enzymes, such as the mitochondrial Lon protease, which can remove oxidized proteins. Another prominent internal source of oxidative stress are peroxisomes, which can generate O_2_^·−^, H_2_O_2_ (and, thus, HO^·^) as a consequence of fatty-acid metabolism. Fortunately, the O_2_^·−^ can be dismutated by CuZnSOD. And the H_2_O_2_ can be removed by catalase which is in extremely high concentration inside peroxisomes. The endoplasmic reticulum also contributes to cellular levels of reactive oxygen species, due to H_2_O_2_ generation from protein folding and the formation of O_2_^·−^ by membrane-bound NADPH oxidase (NOX) enzymes. An additional safety mechanism the cell relies upon is the 20S proteasome. During periods of oxidative stress, the 19S regulatory caps of the 26 proteasome are sequestered away by HSP70 and ECM29, increasing the available pool of ATP-independent 20S proteasomes. These free 20S proteasomes can degrade oxidized proteins, thus preventing their aggregation and cross-linking and providing a pool of (undamaged) amino acids for the synthesis of additional protective enzymes. Overall, a combination of protective cellular mechanisms minimizes damage accumulation and help to maintain cellular homeostasis (depicted by the green background). Thus, in young cells and organisms generally, only a small amount of damage occurs and most of that is repaired. The green balls inside the mitochondria represent small amounts of mildly oxidatively damaged proteins, as do the orange colored balls in the cytoplasm. In both mitochondria and cytoplasm, the yellow star-bursts indicate severely damaged, aggregated and often cross-linked proteins. Lower (light red background) panel—old cells—with age, gradual damage accumulation can lead to less-than-optimal defense systems. As a result, background inflammation and oxidative stress can increase from both external and internal sources (symbolized by the red background). Additionally, the internal generation of O_2_^·−^ and H_2_O_2_ may increase, but the enzymatic defenses, such as the mitochondrial Lon protease or superoxide dismutase may decrease in clearance efficiency, further exacerbating damage accumulation. Similarly, the defense enzymes necessary to protect against peroxisome and endoplasmic reticulum O_2_^·−^and H_2_O_2_ generation may decrease in efficiency with age, together leading to a cellular imbalance toward oxidative damage. Finally, the 20S proteasome must deal with more and more oxidized proteins (indicated by a great increase in green and orange balls, and yellow star-bursts compared with the upper panel of young cells), many of which aggregate and cross-link before they can be degraded. This results in proteasome becoming irreversibly bound to ‘clumps’ of damaged proteins that it cannot degrade. Proteasomes bound to such oxidized protein aggregates (sometimes called lipofuscin) become inactive, thus further decreasing clearance of damaged proteins and enabling ever-increasing protein aggregation

Cellular homeostasis is constantly challenged by reactive oxygen and nitrogen species which are either generated endogenously during cellular metabolism (e.g., by mitochondria, NADPH oxidases, and cytochrome P450 reductases) or originate from exogenous sources including drugs, xenobiotics metals, and radiation. Reactive oxygen and nitrogen species alter a wide variety of macromolecules including proteins, lipids, sugars, DNA, and RNA. Depending on the severity of oxidatively generated damage, newly oxidized molecules are repaired or are eliminated by dedicated degradation systems. When production or clearance of reactive oxygen and nitrogen species is overwhelming or when repair/degrading systems for damaged molecules are impaired, oxidative stress occurs. Excessive amounts of (unrepaired) oxidant-induced cellular damage are associated with cancer, ageing, and numerous other pathologies such as Parkinson and Alzheimer diseases (Beckman and Ames 1998; Stadtman and Berlett 1997). Thus, the first paradox to be considered in this discussion is the “Oxygen Paradox”—the discovery that although oxygen is essential for multicellular eukaryotic life forms, its very chemical/physical nature ensures its conversion into oxygen radicals, and other reactive oxygen species, that cause damage to cells, organs, and organisms. In human terms, it would be very hard to live without oxygen, but it is also extremely hard to live with oxygen (Fig. 1) (Davies and Ursini 1995).

The French paradox was proposed in 1992 based on epidemiological data from the French population (Renaud and de Lorgeril 1992). Diets with high intakes of dietary cholesterol and saturated fat were found to coincide with low rates of coronary heart disease and low risk of cardiac mortality in the French populace. These findings were counterintuitive and were aptly dubbed the “French Paradox.” However, in-depth research has provided new evidence as to why the “French Paradox,” which seemingly is harmful, may actually promote the health and longevity of the French people. For example, the high consumption of wine and cheese, both of which are often viewed in a negative light, may actually be beneficial. Due to wine’s high concentration of resveratrol (Catalgol et al. 2012), and the high prevalence of spermidine in cheese (Eisenberg et al. 2016), the French diet may actually contribute to the positive effects of the French Paradox. Clearly, much of what underlies the French Paradox is, in turn, caused by the “Oxygen Paradox” wherein oxidative stress is seen as an unavoidable consequence of aerobic life (Davies and Ursini 1995) and a major contributor to the initiation and/or progression of many age-related human diseases.

The goal of this review is to explore new viewpoints that may help to explain how basic aspects of the Oxygen Paradox are impacted by the diet and environment and, in turn, affect the etiology and progression of age-related human diseases.

## The role of adaptive homeostasis in the Oxygen Paradox

The third (apparent) paradox to be considered in this review is the seemingly paradoxical observation that treatment with low doses of a substance that is toxic at high concentrations may actually induce transient adaptations that protect against a subsequent exposure to the same (or similar) toxin. Thankfully, we can now mechanistically explain this particular ostensible paradox as the phenomenon of “Adaptive Homeostasis” (Eisenberg et al. 2016; Davies 2016; Pomatto et al. 2017a; Lomeli et al. 2017; Pomatto and Davies 2017),;the non-damaging process by which an apparent toxicant can activate biological signal transduction pathways to increase expression of protective genes, by mechanisms that are completely different from those by which the same agent induces toxicity at high concentrations. Adaptive pathways allow cells and organisms to cope with transient environmental and internal perturbations. It is now clear that resistance to multiple forms of stress is not a static property of cells, tissues, or organisms. Indeed, multiple protective systems demonstrate great transient plasticity in response to very small changes in reactive oxygen species, such as the superoxide anion radical (O_2_^·–^) and hydrogen peroxide (H_2_O_2_). The term Adaptive Homeostasis describes this important cellular capability. Single or repeated exposures to mild stress at younger ages are beneficial against higher, more toxic oxidant levels, indicating that rescue systems are actively induced in the young (Eisenberg et al. 2016; Davies 2016; Pomatto et al. 2017a; Lomeli et al. 2017; Pomatto and Davies 2017). Adaptive Homeostasis has been defined as follows: “The transient expansion or contraction of the homeostatic range in response to exposure to sub-toxic, non-damaging, signaling molecules or events, or the removal or cessation of such molecules or events.” This definition allows us to predict and explain the transient expansion of the homeostatic range that occurs upon exposure to nanomolar, even picomolar, levels of agents that would only be damaging or toxic in the millimolar range. Since a wide variety of diets, environmental conditions, and human activities (including exercise, a pro-oxidant activity) involve both mild stresses and positive health benefits, there is real hope that new strategies relying on adaptive homeostatic effects could be used as palliative or preventive agents in human beings (Rattan and Le Bourg 2014).

**Fig. 2:**
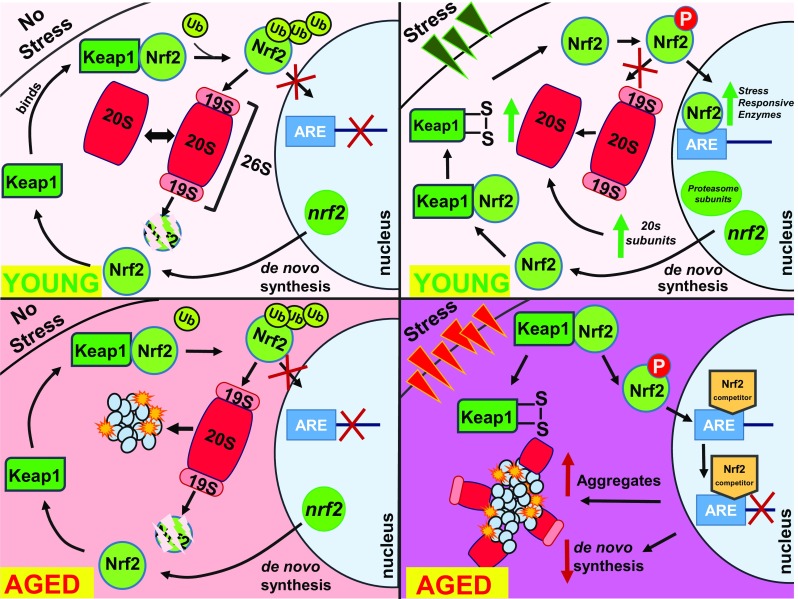
Adaptive homeostasis and nrf2 dependent stress responses decline with age. During cellular homeostasis in young organisms and tissue, nuclear factor (erythroid-derived 2)-like 2 (Nrf2), the master stress-responsive transcriptional activator, is retained in the cytosol bound to the Keap1-Cul3 complex, which contains a ubiquitin E3 ligase. In the absence of stress in young cells (top left panel) Nrf2 is polyubiquitinylated, tagging it for degradation by the ATP-dependent 26S Proteasome, and preventing Nrf2 translocation into the nucleus. Concurrently, as the Nrf2 protein undergoes rapid turnover, the *nrf2* gene is constantly transcribed and translated, and de novo Nrf2 is bound to Keap1-Cul3 complexes, enabling cells to have a constant supply of Nrf2. At the same time, cells retain sizeable pools of both 20S and 26S forms of the Proteasome for (different forms of) protein clearance. During periods of oxidative stress in young cells (top right panel), Nrf2 is released from the Keap1-Cul3 complex and phosphorylated, and then translocated into the nucleus. Once in the nucleus, Nrf2 binds to the antioxidant response element (ARE), also called the electrophile response element (EpRE), leading to the transcriptional upregulation of stress responsive enzymes, including the 20S Proteasome. With age, however (bottom left panel), the ability to mitigate damage declines, resulting in an overall increase in baseline inflammation (symbolized by the pink background). In addition, the pool of available Proteasomes (both 20S and 26S) is diminished, as a certain percentage become inactive after binding to indigestible protein aggregates. The inability to clear away all cellular damage promotes the accumulation of protein aggregates, thus further diminishing the available pool of Proteasome. During periods of acute stress in aged cells (indicated by the darker pink background in the bottom right panel), cellular limitations of the stress-response system become evident, as binding of Nrf2 to ARE/EpRE sequences diminishes. Decreased binding efficiency of Nrf2 to ARE/EpRE sequences may be due to Nrf2 competitors such as c-Myc and Bach1. The net result is significantly decreased ability to rapidly upregulate target stress-responsive genes, further promoting protein aggregation, diminished proteostasis, and ineffective adaptive homeostasis

The importance of adaptive homeostasis, and its decline with age, can be seen in two studies conducted in the fruit fly *Drosophila melanogaster*. Proteasome, a protease involved in the degradation of both normal ubiquitinylated proteins and mutated or damaged proteins, regulates a vast number of cellular functions (Bonet-Costa et al. 2016; Johnston-Carey et al. 2015). Impaired proteasome function promoted several “old-age” phenotypes and markedly reduced the lifespan of flies (Tsakiri et al. 2013a, b; Pomatto et al. 2017b). Proteasome expression is regulated by the NFE2-related factor 2 (Nrf2)/Ketch-likeECH-associated protein 1 (Keap1) signaling pathway. This pathway and its constituent components (i.e., the Nrf2 ortholog, cap’-n’-collar isoform-C (CncC), and the Keap1 and Maf proteins) are conserved in *Drosophila* and appear to engage in similar regulatory interactions as in vertebrates (Sykiotis and Bohmann 2008). The Nrf2 pathway works well in young flies, where it induces proteasome expression and increased oxidative stress resistance. This important mechanism of adaptive homeostasis is severely abrogated in old age. Interestingly, inducible activation of Nrf2 in transgenic flies upregulated basal proteasome expression and activity, independently of age and conferred resistance to proteotoxic stress. Prolonged Nrf2 overexpression, however, reduced longevity indicating that excessive activation of the proteostasis pathways can be detrimental (Fig. 2) (Tsakiri et al. 2013b).

Another cellular stress-responsive protein is the mitochondrial Lon protease. Lon is a major regulator of mitochondria physiology, regulating and chaperoning imported proteins into the mitochondria and degrading unfolded, oxidized, and non-assembled proteins. Acute stressors such as heat shock and oxidative stress lead to its up-regulation (Ngo et al. 2013; Pinti et al. 2010, 2011, 2016). However, prolonged oxidative stress can cause Lon down-regulation (Pinti et al. 2015). A new study by Pomatto et al. (Pomatto et al. 2017c) showed that young female fruit flies (but not males or old flies) were capable of H_2_O_2_ stress adaptation and increased Lon expression, whereas only young males were capable of paraquat stress adaptation and adaptive increase in Lon expression. These results suggest both an age- and sex-dependent threshold for transient oxidative stress adaptation, including increased Lon expression, that is beneficial for survival of a short stress, but costly for longevity. Thus, the balance of positive and negative effects of adaptive homeostasis on fitness traits could vary. Further investigation is necessary to determine what conditions/treatments might ultimately lead to beneficial therapeutic interventions for humans.

A novel family of peptides, encoded within the mtDNA, has been shown to normalize mitochondrial function and to prevent metabolic deterioration, vascular disease, diabetes, and neurodegeneration, namely the mitochondrial-derived peptides, humanin (Yen et al. 2013), MOTS-c (Lee et al. 2016a), and SHLPs (Cobb et al. 2016). These mito-hormones hold promise as both diagnostic and therapeutic agents in human disease and their role in the paradox is being studied.

### The aged proteome and decline of adaptive homeostasis in ageing

Dysregulation of protein homeostasis and accumulation of oxidatively modified proteins by reactive oxygen/nitrogen species and various processes related to the formation of advanced glycated/lipid peroxidation end products (AGEs and ALEs) are hallmarks of the ageing process in different organs and tissues across different species (Stadtman and Berlett 1997; Baraibar and Friguet 2013). In addition, cellular protein maintenance systems, such as the proteasome system, are often themselves affected during ageing and upon oxidative stress, losing effectiveness over time (Baraibar and Friguet 2013). Thus, the accumulation of altered proteins during ageing is due to both an increased production of modified proteins and a decreased efficacy of the mechanisms responsible for their removal (Petropoulos and Friguet 2006).

Among the many types of oxidative protein modifications described, carbonylation is one of the most prominent. Protein carbonylation is irreversible and often leads to loss of function or gain of toxic function of the targeted proteins (Baraibar and Friguet 2013). However, the key question, whether protein carbonylation is directly involved in ageing and age-related diseases, remains unanswered. In recent years, different studies have provided evidence that the oxi-proteome (the build-up of carbonylated proteins) during ageing and age-related diseases is composed of only a limited group of proteins (Baraibar and Friguet 2013), indicating that not all proteins have the same susceptibility for oxidation. For example, the replication and differentiation capability of adult muscle stem cells (satellite cells) is compromised with age, contributing to the development of sarcopenia. However, the molecular events related to satellite cells dysfunction during ageing are not completely understood.

### Decline of adaptive homeostasis in mitochondria

Recent evidence indicates that expression of the Lon protease may be regulated by pro-oxidants in a manner consistent with the removal of oxidatively modified proteins (Ngo et al. 2013). An alternative or additional mode by which Lon may be inactivated or activated in response to pro-oxidants is through its own modification. While free radicals have long been viewed as damaging species, it is now recognized that, by virtue of their reversible nature, certain oxidative modifications to proteins may serve to regulate their function(s).

At the interface between the body and the environment, the skin is directly exposed to numerous chemical oxidants and other air pollutants, all of which are potent inducers of reactive oxygen/nitrogen species (Mancebo and Wang 2015; Valacchi et al. 2012). An in vitro model using a direct exposure device for studying the cellular effects of five VOCs (hexane, toluene, acetaldehyde, formaldehyde, and acetone) at doses mimicking low-dose chronic environmental exposure on skin keratinocytes and skin explants assessed Lon protease activation and degradation of oxidatively modified proteins induced by VOCs exposure. Exposure of cells or skin explants to VOCS induced free radical production that mediated apoptosis due to DNA damage, mitochondrial membrane potential collapse, lipid peroxidation, and proteasome and Lon protease inactivation in keratinocytes and in human skin explants.

It is well known that proteasome dysfunction is a consequence of oxidative stress and that proteasome inhibition induces mitochondrial dysfunction (Livnat-Levanon et al. 2014). In keratinocytes, exposed to mild air pollutants, no induction of Lon expression was observed, suggesting that the damage was so great that VOC induced irreparable injuries which did not allow induction of cellular defenses. Another example assessing the consequence of chronic oxidative stress relies upon the Parkinson Disease mouse model of 1-methyl-4-phenyl-1,2,3,6-tetrahydropyridine (MPTP) intoxication. Bulteau et al. showed that Lon protease expression increased in the ventral mesencephalon of intoxicated animals, concomitantly with the appearance of oxidized proteins and dopaminergic cell loss. In addition, they reported that Lon protease is inactivated by oxidants and is not able to cope with the accumulation of oxidized proteins produced in the mitochondria. In this case, even induction of increased Lon expression is not sufficient to overcome the oxidative insult.

Taken together, these data underscore the importance of adaptive homeostasis. It is increasingly apparent that pro-oxidants do not simply cause damage, but can regulate protein function in a reversible fashion. Thus, critical factors that shift the balance from non-damaging and reversible signaling, to oxidative stress and irreversible loss are major threats to normal function and healthy ageing.

## Heart and circulatory system

The term cardiovascular disease (CVD) actually encompasses an array of diseases including coronary artery disease, hypertension, congestive heart failure, and stroke. Atherosclerosis that begins early in life and progresses gradually is a major cause of cardiovascular disease (Madamanchi et al. 2005).

### Atherosclerosis

The development of atherosclerosis is a complex process, involving the interaction of a variety of cells within the vessel wall, including endothelial cells, trans-differentiated vascular smooth muscle cells, and monocytes/macrophages. These two later cell types are believed to play a pivotal role in the development of the disease, mainly through the accumulation of oxidatively modified low-density lipoproteins (oxLDL, a proatherogenic particle detected in the intima of the arterial wall), and the production of inflammatory mediators, cytokines, and extracellular matrix-degrading enzymes (Glass and Witztum 2001). If left unchecked, will eventually lead to the formation of a lipid core of advanced atherosclerotic plaques (Tabas 2005).

Fortunately, ATP-binding cassette (ABC) transporters maintain cellular cholesterol homeostasis and protect arteries from atherosclerosis (Schmitz et al. 2006). High-density lipoproteins (HDLs), lipoproteins-containing apolipoproteinA1 (apoA1), are the principal lipoproteins that play a major role in RCT.

HDL particles and their apoA1, recovered from human atheroma, are extensively oxidized by myeloperoxidase (MPO) and are non-functional (Huang et al. 2014). In vitro oxidation of either apoA1 or HDL particles by MPO impairs their cholesterol acceptor function. In addition, oxindolyl alanine (2-OH-Trp) moiety at Tryptophan72 (Trp72) of apoA1 is selectively oxidized by MPO, is markedly enriched within human atheroma, and is functionally inactive, including inhibition of the ABCA1-dependent cholesterol acceptor activity of apoA1. OxTrp72-apoA1 recovered from human atheroma or plasma is lipid poor, virtually devoid of cholesterol acceptor activity and demonstrates both a potent pro-inflammatory activity on endothelial cells and an impaired HDL biogenesis activity in vivo. Elevated levels of the MPO-oxidized apoA1 form, oxTrp72-apoA1, in subjects are thus associated with increased cardiovascular disease risk, and may serve for monitoring a pro-atherogenic process in the artery wall. Their studies also reveal MPO-catalyzed oxidation of apoA1 as a potential therapeutic target for the treatment and prevention of atherosclerotic heart disease.

Among the antioxidant systems, thioredoxin-1 (Trx-1), a 12-kDa highly conserved protein, has recently been recognized as a critical protective system against oxidative stress. Interestingly, this protein decreases dramatically with risk factors (Forstermann 2008). The C-terminal truncated form made up of 1–80 or 1–84 N-terminal amino acids (Trx-80), both cleaved by two alpha-secretases (ADAM-10 and ADAM-17) at the cell surface, possesses a cytokine like activity (Pekkari and Holmgren 2004). Macrophages have been reported to cleave Trx-1 to yield Trx-80 (Bizzarri et al. 2005).

In atherosclerosis, Trx-1 polarized macrophages to the M2 anti-inflammatory phenotype and protected the ApoE2.Ki mouse against atherosclerosis (El Hadri et al. 2012). Conversely, Trx-80, oriented macrophages toward the M1-pro-inflammatory phenotype and enhanced atherosclerosis in the same animal model (Mahmood et al. 2013), promotes pro-inflammatory macrophages of the M1 phenotype and enhances atherosclerosis. Trx-80 also exerts angiogenic and mitogenic effects (Couchie et al. 2017). In addition, isolated perfused hearts adapted to ischemia and reperfusion by short cycles of ischemia and reperfusion (I/R), the protection is apparently afforded by the enhanced induction of Trx-1 (Turoczi et al. 2003). Further studies conducted in animal models suggest that Trx-1 has many beneficial functions in the heart including a protective role in cardiac hypertrophy (Matsushima et al. 2011; Yamamoto et al. 2003).

Endothelial and vascular smooth muscle cells (VSMC) are involved in the vascular ageing and play a major role in CVD through the endothelial dysfunction and VSMC transdifferentiation/calcification. The senescent phenotypes associated with Lamin A/C alterations can be due to mutations in the lamin-encoding gene LMNA resulting in familial lipodystrophy FPLD2 associated with early atherosclerosis (Bidault et al. 2013), or treatment with some anti-HIV antiretroviral drug (ART), such as protease inhibitors (PI) (Lefèvre et al. 2010). These treatments are associated with increased cardiovascular risk in ageing HIV-infected ART-controlled patients and alter the processing of prelamin-A to lamin-A resulting in the accumulation of farnesylated prelamin-A leading to cell senescence. By comparing the endothelial function and senescence in primary human coronary endothelial cells culture issued from an old and a young individual, old subjects accumulated prelamin-A and exhibited endothelial dysfunction, oxidative stress, inflammation, and senescence. Most PIs induced the accumulation of farnesylated prelamin-A, as a result of their ability to inhibit the enzyme involved in the processing of prelamin-A to lamin-A, ZMPSTE24 as well as endothelial dysfunction, oxidative stress, inflammation, and senescence. Importantly, statin treatment prevented all those alterations; other classes of ART molecules, such as maraviroc or dolutegravir, entry or integrase inhibitors, respectively, were either not affecting vessels or able to decrease inflammation and senescence.

Overexpressing mutant LMNA in primary human VSMC culture or treating them with PIs increased prelamin-A and decreased ZMPSTE24. Similar results were obtained in fibroblasts from LMNA-mutated lipodystrophic patients and peripheral blood mononuclear cells from PI-treated-HIV-infected patients. These alterations positively correlated with oxidative stress, inflammation, senescence, and calcification. ZMPSTE24 silencing in native VSMCs recapitulated the mutant LMNA- and PI-induced accumulation of farnesylated prelamin-A, oxidative stress, inflammation, senescence, and calcification. The farnesylation inhibitors pravastatin and the farnesyl-transferase inhibitor, FTI-277, or the antioxidant N-acetyl cysteine, partly restored ZMPSTE24 expression, and concomitantly decreased oxidative stress, inflammation, senescence, and calcification of PI-treated VSCMs. Thus, ZMPSTE24 down-regulation, resulting from LMNA mutations or PI treatments, should be considered as a major contributor in VSMC dysfunctions in early atherosclerosis. Statin and antioxidants could be of therapeutic interest in that setting.

VSMC change of phenotype (i.e., their transdifferentiaton) contributes to the pathogenesis of age-related CVD such as atherosclerosis and post-angioplasty restenosis. Cyclic AMP, the production and degradation of which are catalyzed by adenylyl cyclases (AC) and phosphodiesterases (PDE), respectively, plays a key role in modulating VSMC phenotype. Additionally, transdifferentiation of VSMCs into inflammatory/migratory cells in vitro depends on de novo expression of the transmembrane AC isoform 8 (Clement et al. 2006). The pathological VSMCs in human atherosclerosis lesions have high AC8 levels (Gueguen et al. 2010).

### Stroke

Age is a well-documented prominent non-modifiable risk factor for stroke, with arterial stiffness as a major independent determinant of increasing systolic and pulse pressure and a tissue biomarker of cardiovascular risk factors. It is becoming increasingly clear that VSMC plasticity significantly contributes to arterial stiffening in ageing (Lacolley et al. 2012; Sehgel et al. 2015). The process of phenotypic switching requires a specific pattern of transcriptional factors, in particular the SRF and myocardin, and microRNA expression whose mechanisms are now beginning to emerge. Using the conditional SMC-inactivation of SRF gene mouse model, SRF controls pressure-induced myogenic tone and vasoconstriction in mesenteric arteries via VSMC phenotypic modulation linked to changes in contractile protein gene expression (Galmiche et al. 2013). SRF-related decreases in vasomotor tone and cell-matrix attachment increase arterial elasticity in large arteries. By contrast, in mice with inducible SRF overexpression, the in vitro contractile response was significantly increased in all arteries. Using the knock-out (KO) mouse model of intermediate filament genes such as desmin and vimentin, they have shown that a lower distensibility and an increase of arterial wall viscosity in desmin KO mice (Lacolley et al. 2001), and vimentin ablation results in decreased arterial pressure and increased carotid wall stiffness (Langlois et al. 2017). They observed that several endothelial markers are up-regulated and content of several components of basement membrane such as laminin, fibronectin, and perlecan is increased in vimentin KO mice, suggesting a role of endothelial cells in vascular stiffness.

### Congestive heart failure

As stated above, CVD also encompasses congestive heart failure, a morbid disorder whose prevalence strongly increases with the ageing of the world population and the spread of Western dietary pattern. Despite continuous progress in the therapy of heart failure, which is mainly based on the use of β-blockers, neurohormonal antagonists, and diuretics, morbidity and mortality remain high for this disease and the economical and societal costs keep rising. One major issue that is poorly addressed in current heart failure therapy is the severely depressed bioenergetics functions in the failing heart. Indeed, the failing heart is characterized by deficient mitochondrial functions leading to a decrease in high-energy phosphate metabolites levels (ATP, phosphocreatine) and an increase in oxidative stress. Nicotinamide adenine dinucleotide (NAD+) is a major hydride-transfer coenzyme in the process of glucose and fatty acids oxidation and a major electron donor to the mitochondrial electron transport chain allowing the synthesis of ATP. NAD(H) is the sole precursor of NADP(H) which has a dual role, either acting as the final donor of electrons in oxygen radical detoxification systems (glutathione, thioredoxin, etc.) or participating in the generation of superoxide by NADPH oxidases in oxidative burst reactions.

Myocardial NAD+ level decreases in the failing heart of many different mouse models of heart failure (Mericskay 2016). This decrease parallels the down-regulation of the major NAD+ salvage pathway mediated by the NAMPT enzyme in most forms of heart failure (Hsu et al. 2014). Supplementation of food with NAD precursors can markedly improve cardiac functions while preserving cardiac energy metabolism and allowing the cardiac cells to cope with pathological changes to the redox state. These findings (Pillai et al. 2005; Yamamoto et al. 2014; Lee et al. 2016b) suggest that maintaining NAD levels in the heart constitute a new therapeutic avenue for the treatment of heart failure, a syndrome strongly associated with ageing.

To address the issue of heart tissue injury, a new approach based on cardiac tissue engineering for heart failure treatment has been developed. The principle of tissue engineering mainly relies upon the promotion of cells growth on a bio-inspired scaffold that mimics both physical and chemical properties of cardiac tissues (Kitsara et al. 2016). To develop a new generation of cell therapy products made of cellularized biomaterials, robust differentiation protocols were developed to generate contractile human cardiomyocytes derived from pluripotent stem cells (Joanne et al. 2016). Different biomaterials composed of nanofibers from extracellular matrix components were developed (i.e., collagen, hyaluronic acid) or natural polymers (i.e., fibrin, chitosan) mimicking the alveolar and fibrous structure of the myocardium (Joanne et al. 2016; Bellamy et al. 2015; Fiamingo et al. 2016). Moreover, these biocompatible and biodegradable biomaterials could be seeded with human cardiomyocytes, enabling the development of functional cardiac-like tissue. Finally, engraftments of these cellularized biomaterials on the surface of the heart demonstrate integration and functionality of engineered tissues in animals (Joanne et al. 2016). In conclusion, combining cardiac cells with the biomaterials to control both biological and physical-chemical environments of the cells allows the development of new cell therapy products for cardiovascular diseases.

Based on previous reports of a decreased ability to activate the Nrf2 pathway in animal studies (Zhang et al. 2012), it was hypothesized that the ability to activate Nrf2 would decline in primary human cells with age. Primary human bronchial epithelial (HBE) cells were obtained from six different male, non-smoking subjects (three from age 28–29 and three from ages 67–69). Three comparisons were made by randomly pairing one young with one old into groups. Cells of low passage were treated with or without 2 μM sulforaphane for 12 h, and the mRNAs of Nrf2-regulated antioxidant (phase II) genes were measured. Unstimulated expression of GCLC, GCLM, HO1, and NQO1 genes was the same in the young and old HBE. In contrast, sulforaphane-induced phase II gene expression of the young HBE cells was significantly greater than that in the older cells supporting our hypothesis. Western blot analysis showed an increase in Nrf2 accumulation within 1 h in cell nucleus after stimulation with sulforaphane in both young and old cells, suggesting that the difference in response occurs in the nucleus. Bach-1 and c-Myc which are Nrf2 suppressors were subsequently quantified at baseline and after stimulation. The results mimic what has been observed in several previously reported studies of animal organs.

## Myocardial ischemia and reperfusion injury

The management of myocardial infarction has evolved dramatically over the last 30 years. In the 1980s, reperfusion therapies were developed, first with thrombolytics, and now with primary percutaneous coronary interventions. It is well accepted that acute reperfusion with angioplasty, thrombectomy, and stenting is the current state-of-the-art treatments. Early reperfusion therapy with percutaneous coronary interventions (stents and/or balloon angioplasty) and/or thrombolytic therapy has reduced the size of myocardial infarctions and improved survival. However, despite falling door-to-balloon times, mortality rates have plateaued. According to the American Heart Association, about 15% of patients who experience a myocardial infarction in any given year will die of it. While 30-day mortality rates still were reported to be 7.8 to 11.4% in some analyses (Mozaffarian et al. 2015). Nearly a quarter of older patients suffering a myocardial infarction will suffer congestive heart failure after 1 year. Therefore there is an unmet need to further reduce structural and functional damage to the heart following a myocardial infarction.

One approach is to further reduce myocardial infarction size. It is well recognized that the size of the myocardial infarction is a major determinant of outcome (Boden et al. 2013; Turer et al. 2005). The desired goal is to decrease the myocardial infarction size above and beyond reperfusion, by reducing reperfusion injury. In this review, we will describe the four basic types of reperfusion injury, potential mechanisms of reperfusion injury, attempts to reduce reperfusion injury by scavenging or inhibiting the formation of ROS, and new targets related to reperfusion injury.

In the early seconds to minutes of reperfusion, following a coronary artery occlusion, there is a burst of reactive oxygen species (Zweier et al. 1987). These reactive oxygen species include O_2_^·–^ and H_2_O_2_ that may contribute to membrane damage, increase cell membrane permeability, disrupt ion channels, and allow for calcium overload to the cell.

There are four basic types of reperfusion injury: reperfusion arrhythmias, stunned myocardium, microvascular obstruction (no-reflow phenomenon), and lethal myocardial cell injury (which means that cells that are viable at the end of the ischemic period die because of reperfusion). Reperfusion arrhythmias include ventricular arrhythmias such as ventricular premature beats, runs of ventricular tachycardia, and ventricular fibrillation that may occur very early after reperfusion. These arrhythmias are rarely a major clinical problem and are treated pharmacologically if needed and usually subside over time (Kloner et al. 2011).

### Stunned myocardium

Stunned myocardium refers to previously ischemic viable myocardium that has been salvaged by reperfusion, in which there is prolonged, but transient post-ischemic contractile dysfunction (Braunwald and Kloner 1982). Early experimental studies showed that a brief 15-min coronary artery occlusion followed by reperfusion did not kill myocardial cells; rather these cells showed functional abnormalities and subtle ultrastructural abnormalities that resolved in a few days (Heyndrickx et al. 1975; Kloner et al. 1983). During the coronary artery occlusion, a regional wall motion abnormality was demonstrated within the zone of ischemia; however, following reperfusion and despite the fact that the reperfused myocardium was viable, persistent ventricular wall motion abnormalities persisted for several days (Heyndrickx et al. 1975; Kloner et al. 1983). Hence, the myocardium behaved as if it were stunned. Using ultrasonic crystals implanted into the wall of the left ventricle to track percent segment shortening, Przyklenk et al. (Przyklenk and Kloner 1986) showed that superoxide dismutase and catalase, given prior to, during occlusion and during reperfusion, improved the recovery of the segment shortening within the stunned segment. This finding and those of the Bolli group (Bolli 1988) showed that the mechanism of stunned myocardium involved reactive oxygen species and that a potential therapy for stunned myocardium was oxygen radical scavengers. Another theory regarding stunning was that calcium overload into the cells, due to increased permeability and damage to membranes caused by reactive oxygen species, then desensitized the contractile apparatus to calcium, preventing normal myofilament function. We observed that in similar experimental models that the calcium channel blocker, nifedipine, also improved the function of stunned myocardium (Przyklenk et al. 1989).

Stunned myocardium has been described in patients and includes the following: gradual return of regional function following thrombolytic therapy for acute myocardial infarction; prolonged left ventricular regional wall motion abnormalities in patients with unstable angina, persistent left ventricular regional wall motion abnormalities following exercise-induced ischemia, and prolonged but reversible left ventricular dysfunction following cardiac surgery (Patel et al. 1988). Stunned myocardium does respond to inotropic stimulation (Arnold et al. 1985). In the setting of post myocardial infarction, stunning or post cardiac surgery stunning where heart failure is an issue, inotropes are often used to hold the patient over until the stunning recovers. Because stunning does eventually resolve, by definition, it can usually be managed in the clinical setting using general measures such as inotropes.

### No-reflow phenomenon

The no-reflow phenomenon is the inability to reperfuse regions of the myocardium after re-establishing patency of an occluded epicardial coronary artery. The usual cause is microvascular obstruction. Experimental studies showed that following release of a 90-min or longer coronary artery occlusion in the canine model of myocardial infarction that the markers of flow do not perfuse portions of the subendo-and mid-myocardium, despite the fact that the epicardial infarct artery is patent (Kloner et al. 1974). These zones of no-reflow are characterized by anatomic evidence of microvascular obstruction. Ultrastructural analysis within the zone of no-reflow revealed localized swelling and protrusions of the endothelium that appear to block the lumen. It was also observed the no-reflow phenomenon in other species including rabbit and rat with as little as 30 min of coronary artery occlusion followed by reperfusion. There is evidence that no-reflow is a true form of reperfusion injury. It was observed that size of the no-reflow zone expands over the first few hours after reperfusion of an epicardial coronary artery, suggesting worsening microvascular damage during the phase of reperfusion (Reffelmann and Kloner 2002). In other experimental studies, we observed that no-reflow correlates with worsened left ventricular remodeling during the chronic phase of infarction. Ventricles with no-reflow are likely to demonstrate more thinning and stretching of the infarct wall and left ventricular dilatation (Reffelmann et al. 2003). The most likely explanation for this is that no-reflow inhibits the efflux of cellular debris from the necrotic core and limits entrance of those humoral factors and cells that are important to the healing phase such as macrophages and fibroblasts. Studies by Przyklenk and Kloner showed that oxygen radicals may play a role in the microvascular damage associated with no-reflow. Upon administration of superoxide dismutase and catalase, given just prior to reperfusion after a 2-h proximal coronary artery occlusion, improved regional myocardial blood flow measured with radioactive microspheres in the endo-, mid-, and epicardial layers of the myocardium. In addition, superoxide dismutase and catalase improved the ultrastructural appearance of the microvessels (Przyklenk and Kloner 1989). Of note, these benefits of the oxygen free radical scavengers were independent of any effect on myocardial infarct size, since these drugs did not reduce infarct size in our models. Other experimental therapies that have reduced the no-reflow phenomenon in our myocardial infarct models include therapeutic hypothermia, when administered either early, during the phase of ischemia, in which it also reduces myocardial infarct size (Hale et al. 2003); or when administered late, after reperfusion has already been established, in which case it reduces the no-reflow zone but has no effect on myocardial infarct size (Hale et al. 2013). Finally we recently observed that the mitochondrial targeted peptide, elamipretide, which is known to reduce the generation of reactive oxygen species from the mitochondria during stress, reduced the size of the no-reflow zone for any given risk zone in a rabbit model of coronary artery occlusion (Kloner et al. 2012).

No-reflow has been documented in patients following reperfusion of the epicardial infarct related arteries. Perfusion defects within the myocardium have been observed by contrast enhanced magnetic resonance imaging, echocardiographic contrast injections, and nuclear studies (Kloner 2011). In the catheterization laboratory, low TIMI grades, long TIMI frame counts, and reduced myocardial blush grades and slow resolution of ST elevation on the ECG are other findings consistent with no-reflow (Rezkalla et al. 2010). Patients who demonstrate no-reflow after myocardial infarction also demonstrate more adverse left ventricular remodeling including a more dilated ventricle (Kloner 2011), which parallels the findings described in the experimental literature. Patients who demonstrate no-reflow following a myocardial infarction have worse survival rates than patients without no-reflow and this observation is independent of infarct size. No-reflow in humans may differ somewhat from that described in the experimental models as it also can involve micro-emboli of atherosclerotic and embolic debris released downstream into the microvasculature following coronary balloon inflation and/or stent deployment. While there is no standardized therapy for treating no-reflow in the catheterization laboratory, some of the strategies that have been employed include adenosine, verapamil, nitroprusside, systemic anticoagulation and platelet 2B-3A receptor blockers, aspiration thrombectomy, and distal protection filters (Kloner 2011).

### Lethal myocardial cell injury

Lethal cell injury is the most important, but probably the most controversial, of the forms of reperfusion injury. Here, cells that are reversibly injured at the end of a period of ischemia become irreversibly injured by the act of reperfusion. Presumably reactive oxygen species generation, coupled with calcium overload lead to additional cellular damage at the time of reperfusion. If a drug or other therapy is administered at the time of reperfusion and the size of the myocardial infarction is reduced compared to reperfusion alone, then this finding would support the concept that reperfusion injury exists and that the adjunctive therapy was working by limiting reperfusion injury. While some studies suggest that drugs given at reperfusion can limit the size of a myocardial infarction (Yellon and Hausenloy 2007), other studies show contrary results. These studies have demonstrated that for an adjunctive therapy to limit myocardial infarct size, the drug or therapy had to be on-board during a substantial phase of ischemia or given before ischemia. Thus therapies such as hypothermia (Hale et al. 2003, 2013), remote ischemic conditioning (Przyklenk et al. 1993), calcium channel blockers (Lo et al. 1985), and beta blockers (Rasmussen et al. 1977) which reduced infarct size in some of these models needed to be present prior to or during the ischemic insult. Given only after reperfusion, they did not work. Hence, postconditioning did not reduce myocardial infarction size in these models (Dow and Kloner 2007). Similarly, superoxide dismutase and catalase, given at reperfusion, failed to reduce myocardial infarct size, but did reduce microvascular obstruction (Przyklenk and Kloner 1989). In general, clinical trials reducing infarct size by giving agents at reperfusion have been disappointing (Gerczuk and Kloner 2012). Recent clinical trials involving postconditioning and mitochondrial permeability pore blocking agents (cyclosporine) have been negative (Kloner et al. 2017). An older study, utilizing superoxide dismutase was negative (Flaherty et al. 1994). Some studies showing certain adjunctive agents when given during ischemia, but prior to reperfusion, reduced infarct size in clinical trials including adenosine, remote ischemic conditioning, successful hypothermia in patients with anterior infarcts, hyperoxemia, beta blockade, and others.

In summary, there are several forms of reperfusion injury. Stunned myocardium and the no-reflow phenomenon are well documented in both preclinical experimental models and in patients. Therapies are available for both, but there is a clinical need to better develop systematic therapy for no-reflow. Such therapy has the potential to enhance healing after a myocardial infarction, reduce adverse left ventricular remodeling, reduce heart failure, and improve survival. The concept of lethal myocardial cell injury, due to reperfusion, remains somewhat controversial in our opinion. However, since there is time to administer agents to patients with infarcts prior to reperfusion therapy in the ambulance or emergency room, there is still the potential to reduce myocardial infarct size above and beyond reperfusion.

## Lung injury, oxygen, and acute respiratory distress syndrome

Oxygen and its potential for toxicity has compounded the management of acute lung injury and the acute respiratory distress syndrome (ARDS). Though there are multiple causes for ARDS, the ultimate insult is an injury to the endothelial barrier to the vasculature and leakage of protein-rich fluid into the alveoli. This results in severe hypoxemia and acute respiratory failure. Clinical studies over the past four decades have shown slow progress in the management of ARDS.

In the 1970s, high fraction of inspired oxygen (FIO_2_), volume ventilation, and positive end expiratory pressure allowed for some improvement in maintaining normoxemia (Petty and Ashbaugh 1971). The addition of larger tidal volumes (10–15 cm^3^/kg), in the 1980s and early 1990s, became the standard of care in the ICU management. However, this approach was accompanied by severe life-threatening complications of barotrauma while attempting to find a ”safe” FIO_2_ (Pierson 2013). Failure to lower FIO_2_ usually resulted in worsening lung injury and increased mortality. At the turn of the twenty-first century, the ARDS network confirmed the value of lung protective ventilation with lower tidal volumes (5–6 cm^3^/kg) and airway pressures than in prior decades, resulting in improved survival (Brower et al. 2000).

The avoidance of oxygen toxicity led to reliance on higher levels of positive end-expiratory pressure (PEEP). The reduction in tidal volume continues to persist and remains the oxygen paradox with some boundaries for clinicians but no simple solutions. Though numerous medications including steroids, non-steroidal anti-inflammatory agents, surfactants, and nitrous oxide have been tested. There is no approved medication for ARDS. The next area of study is moving from barotrauma to biotrauma. The one new area of drug research involves stabilizing the endothelial injury with interferon-β (IFN-β) (Traumakine) (Bellingan et al. 2014). The intravenous infusion stabilizes the injury by replacing the loss of CD 73 (5′Nucleatidase). Currently it is in stage 3 clinical trials in Europe and Japan and awaiting final approval.

## Liver and the French Paradox

The liver plays a crucial role in overall human health and is necessary for proper immune function and lipid and carbohydrate metabolism (Rouiller 2013). Loss in hepatic homeostasis can result in debilitating conditions, one of the most prevalent being nonalcoholic fatty liver disease (NAFLD). NAFLD encompasses a broad spectrum of pathological conditions, ranging from steatosis at the early stages, to nonalcoholic steatohepatitis (NASH) and cirrhosis as the disease progresses. The early stages of the disease are characterized by the accumulation of triglycerides (TAG) in over 5% of hepatic cells, resulting in not only ectopic fat accumulation, but the negative repercussion of low-grade chronic inflammation resulting in tissue normally not accustomed to lipid accumulation (Fabbrini et al. 2010). Eventually, if not corrected, NAFLD can lead to liver failure (Musso et al. 2011) and/or hepatocellular carcinoma (Marrero et al. 2002).

Currently, in the USA, over 30% of adults will develop NAFLD, with this percentage predicted to rise with age (Browning et al. 2004). The increase in disease prevalence is largely attributed to excess caloric consumption, largely resulting from calorie-dense westernized diets. In turn, excess caloric intake not only promotes NAFLD, but loss in blood glucose regulation. Hence, it is of little surprise that the rise in NAFLD coincides with the increasing development of the metabolic syndrome, which is a generalized term for metabolic abnormalities, including increased abdominal fat, insulin resistance, and high blood pressure and blood triglycerides (Alberti et al. 2005). NAFLD stems from hepatic TAG generation exceeding the rate of hepatic TAG catabolism (Birkenfeld and Shulman 2014). Indeed, as early as 3 days following consumption of high fat-diets can induce hepatic insulin resistance in mice and rats (Samuel et al. 2004). Thus, NAFLD has been aptly coined as the hepatic manifestation of the metabolic syndrome (Lebovics and Rubin 2011).

Unless corrected, metabolic complications can increase the risk of developing type II diabetes mellitus (T2DM), predominantly characterized by loss in insulin sensitivity (insulin resistance), and is a disease strongly associated with NAFLD. Moreover, the prevalence of individuals with T2DM and NAFLD is almost double the amount found in age-matched healthy controls, ranging from 50 to 75% (Targher et al. 2007). Consequently, these individuals are at a greater risk for developing more advanced forms of NAFLD, and ultimately, increased risk of mortality (Ong et al. 2008).

Paradoxically, the French diet, which is primarily known for its rich diet (i.e., foods high in saturated fats), which are known contributors of chronic conditions, including NAFLD and coronary artery disease, yet strikingly shows a relatively low prevalence in the French population (Gambini et al. 2015). This has been attributed to the relatively high consumption of red wine, characterized for its highly beneficial components, including polyphenols, which is the staple drink in the French diet. One of the most well-characterized components of red wine is resveratrol and has been linked to numerous beneficial health improvements (Yang and Lim 2014). Indeed, studies in mice fed a high fat diet supplemented with red wine showed improved glucose tolerance, insulin sensitivity, and decreased hepatic steatosis. Moreover, when the same animals were fed the “waste” product of the wine extraction, pomace, caused the greatest improvements in these markers (Rosenzweig et al. 2017). However, it is important to note that excess alcohol consumption can have detrimental effects, and individuals should practice moderation in the extent and type of alcohol that they consume.

## Cancer and carcinogenesis

Among the main non-communicable diseases, cancer represents the leading cause of death worldwide, with approximately 8.2 million deaths and 14 million new cases in 2012 (Stewart and Wild 2014). Western populations are the most affected, especially in France and in the USA, with an age-standardized rate for all cancers higher than 300 cancers per 100,000 people in 2012 (Ferlay et al. 2015). When diagnosed, several therapies such as chemotherapy, radiation therapy, or surgery (or multiple forms of combination therapies) are used to treat cancer, depending on type, localization, and grade. In addition, patients’ diet and physical activity levels are known to modulate the efficiency of these anticancer treatments, but the molecular mechanisms remain unknown (Friedenreich et al. 2010; Davies et al. 2011). Oxidative stress is a key factor in both carcinogenesis and response to anticancer therapies, increasing the interest in reactive oxygen species-targeted therapies (Gorrini et al. 2013; Gentric et al. 2017; Batista et al. 2013; Costa et al. 2014). Among them, physical activity and antioxidant supplementation have progressively become recognized as key actors in preventing cancer development (Gueritat et al. 2014; Rebillard et al. 2013). We address new insights on the controversial role of reactive oxygen species in carcinogenesis and in responses to chemotherapy and/or adjuvant therapies. Moreover, we elaborate on the benefits of individualizing the treatment according to the tumor redox status.

### The double face of reactive oxygen species in cancer

Altered redox balance is an important hallmark of cancer. In 2009, Luo et al. added on the list of common cancer characteristics a new set of hallmarks called “Stress phenotype of cancer”. Among these are DNA damage, mitotic stress, metabolic stress, and oxidative stress, which are all sensitive to reactive oxygen species-mediated signaling (Luo et al. 2009). The levels of oxidatively damaged DNA (i.e., 8-oxo-7,8-dihydroguanine (8-oxoGua)), lipid peroxidation adducts [i.e., 4-hydroxy-2-nonenal (4-HNE) and malondialdehyde (MDA)] and/or protein carbonyls increase in blood samples, urine excretion, and tumor tissue of patients with breast, colorectal, lung, prostate, esophageal, and thyroid cancer (Sheridan et al. 2009; Crohns et al. 2009; Lagadu et al. 2010; Young et al. 2010; Kosova et al. 2014). Some studies also reported that the levels of oxidatively induced damage correlates with poorer survival or even with more advanced stages of the disease (Crohns et al. 2009; Dziaman et al. 2014). Accordingly, cancerous tissues have been found to produce elevated levels of H_2_O_2_, compared to adjacent normal tissues (Szatrowski and Nathan 1991). In the same way, polymorphisms in antioxidant genes are linked to increased cancer incidence (Freriksen et al. 2014). Finally, resistance to reactive oxygen species-producing chemotherapeutic agents is directly proportional to the cellular antioxidant capacity of the patients (Ramanathan et al. 2005).

Reactive oxygen and nitrogen species, mainly produced by xanthine oxidase (XO), NADPH oxidases (NOX), nitric oxide synthase (NOS), and inducible nitric oxide synthase (iNOS) and mitochondria in cancer cells (Huang et al. 2015; Roy et al. 2015; Panieri and Santoro 2016), play a dual role in cancer, depending on the levels generated within the cell or in the microenvironment. Reactive oxygen species, particularly the hydroxyl radical (·OH) induce DNA mutations that might lead to tumor initiation and promotion by targeting tumor suppressor and pro-oncogenic genes (Higinbotham et al. 1992). Together, oxidatively generated DNA damage, coupled with a defective repair system, are widely accepted as important mutagenic factors involved in carcinogenesis (Loft and Poulsen 1996). The sustained presence of reactive oxygen species is associated with the development of metabolic perturbations that provide cancer cells with amino acids, lipids, and nucleotides to further boost their uncontrolled proliferation (Sainz et al. 2012). It is important to note, the amounts of intracellular reactive oxygen species are well controlled in cancer cells, depending on the stage: low-to-moderate reactive oxygen species levels are produced during malignancy transformation (Bostwick et al. 2000; Cullen et al. 2003; Sander et al. 2003). Yet, upon cancer progression, reactive oxygen species production is limited, protecting malignant cells from cell death (Sander et al. 2003; Durak et al. 1993; Portakal et al. 2000; Janssen et al. 2000). This redox harmony between antioxidant and pro-oxidant species is the main secret behind cancer progression. The type of tumor, the context of tumor’s microenvironment, and the nature of stimuli will determine if reactive oxygen species will shift the balance toward a pro-survival or pro-death response (Gentric et al. 2017; Costa et al. 2014).

### Chronic oxidative stress improves responses to anticancer treatment: underlying mechanisms identified in aggressive breast cancer and ovarian carcinomas

Women patients affected by triple-negative breast cancers and ovarian high-grade serous carcinomas, the most aggressive form of breast and ovarian cancers, are generally treated with platinum- or taxane-based chemotherapy (e.g., cisplatin, paclitaxel), two powerful generators of both reactive oxygen species and consequent DNA damage (Alexandre et al. 2007). Interestingly, while chronic oxidative stress increases tumor growth and spread, recent studies have demonstrated that high basal reactive oxygen species levels in breast and ovarian adenocarcinoma cells also improve response to reactive oxygen species-producing chemotherapy (Gruosso et al. 2016; Mateescu et al. 2011). These beneficial effects are mediated by increased cancer cell apoptosis (Ramanathan et al. 2005; Mateescu et al. 2011; Batista et al. 2016), modulation of autophagy (Lefort et al. 2014), regulation of MEK pathway (Gruosso et al. 2015), and DNA damage response (Gruosso et al. 2016).

In the last years, Mechta-Grigoriou’s research group identified the microRNA miR-200 family as a new molecular player linking chronic oxidative stress and chemosensitivity (Mateescu et al. 2011; Batista et al. 2016). Many adaptive mechanisms that modulate gene expression are activated in response to oxidative stress, including the p38α mitogen-activated protein kinase (MAPK) family, recognized to suppress tumorigenesis by blocking proliferation or promoting apoptosis (Hui et al. 2007; Wagner and Nebreda 2009). Interestingly, Mateescu and colleagues showed that the acute oxidative stress increases miR-200 family expression (miR-141 and miR-200a) in non-transformed cells, thus inhibiting p38α activity and promoting malignancy (Mateescu et al. 2011). However, while overexpression of miR-200a expression decreases p38α/MAPK14 protein in ovarian adenocarcinomas and promotes tumorigenesis, it enhances tumor cell death and slows tumor growth under treatment with paclitaxel (Taxane family group) (Mateescu et al. 2011). These observations demonstrate the tight relationship between miR-200 family members, oxidative stress, and cell apoptosis that has been validated by many other studies (Batista et al. 2016; Magenta et al. 2011; Xiao et al. 2015). It has been recently confirmed that miR-200c and miR-141 sensitize ovarian cancer cells to paclitaxel, but confer resistance to carboplatin (Brozovic et al. 2015). Interestingly, paclitaxel is a reactive oxygen species-producing chemotherapeutic agent and its efficacy has been directly linked to antioxidant defenses detected in patient blood (Ramanathan et al. 2005). Taken as a whole, these works emphasize the crosstalk between intracellular reactive oxygen species levels in tumors and levels of antioxidant defenses in response to reactive oxygen species-producing therapies, such as paclitaxel.

More recently, Mechta-Grigoriou’s research group identified the histone variant H2AX, a key component in DNA repair, as being degraded by chemotherapy in Triple-Negative (TN) breast cancer patients (Gruosso et al. 2016). Importantly, H2AX decrease is a marker of chemotherapy efficiency. This study newly demonstrates that chronic oxidative stress, due to JunD−/Nrf2-deficiency, promotes degradation of H2AX by the E3 ubiquitin ligase RNF168. Gruosso and colleagues reported that cycles of chemotherapy (Adryamicin/Cyclophosphamide and Taxanes) given to TN breast cancer patients drives an Nrf2-antioxidant response. The extent of this antioxidant response in patients is linked to H2AX maintenance and resistance to chemotherapy. In tumor cells with low Nrf2-antioxidant response after treatment, the levels of reactive oxygen species remain high, H2AX is efficiently degraded, further reducing repair of DNA damage induced by the drug, tumor cell apoptosis is subsequently increased, and patient survival improved (Gruosso et al. 2016).

### Physical exercise and antioxidant supplementation to target reactive oxygen species in tumor: the need to individualize according to cancer grade

During the last decade, studies investigating the impact of physical activity (PA) on cancer have increased exponentially giving birth to a new field named “exercise-oncology.” Mainly motivated by the evidence that PA could decrease cancer incidence, with a linear dose-response relationship in breast and colon cancer (Ballard-Barbash et al. 2012). Emerging data from breast, colon, and prostate cancer indicate that physically active patients exhibited less recurrence and mortality, highlighting a possible role for PA in the reduction of tumor growth (Meyerhardt et al. 2006; Friedenreich et al. 2016).

The ability of exercise to mimic an antioxidant response has been suggested as a mechanism to counteract the role of reactive oxygen species in tumor growth (de Sousa et al. 2016). In a murine model of low-grade prostate cancer, Gueritat and colleagues demonstrated that regular physical training reduced prostate tumor growth (Gueritat et al. 2014). Although intratumoral protein carbonyls and lipid peroxidation breakdown products (i.e., 4-HNE) were unaffected, the levels of oxidatively generated DNA damage (i.e., 8-oxoGua) were significantly reduced by physical activity. These events were probably due to a decrease in intracellular reactive oxygen species in tumor, as the activation of the redox-sensitive kinase ERK1/2 was also attenuated. Accordingly, since ERK1/2 plays an important role in promoting tumor growth, its inhibition by exercise was positively reverberated on the rate of tumor cell proliferation (Gueritat et al. 2014).

**Fig. 3:**
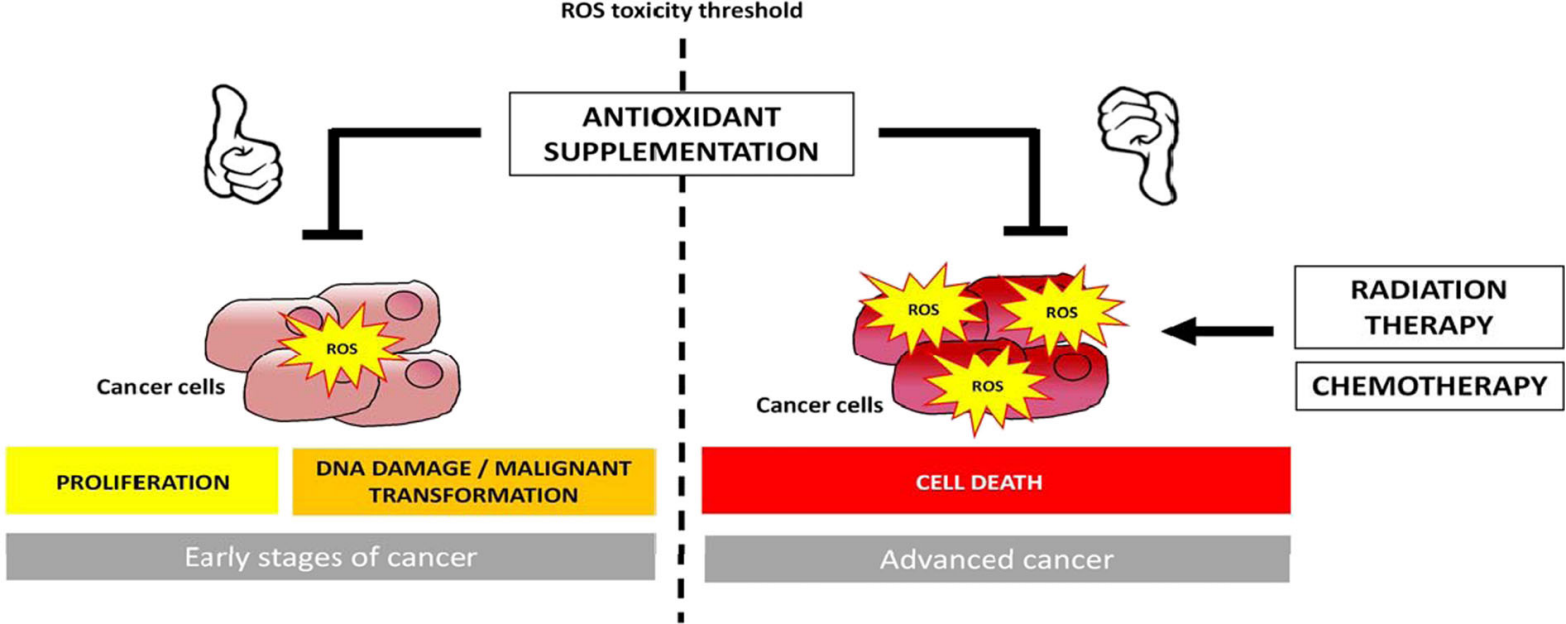
Dual effects of antioxidant supplementation in cancer initiation and development. A combination of preclinical and clinical data provide support for the concept that intervention with nutritional antioxidants might be beneficial at early stages of cancer, but harmful in advanced stages. Early stages of cancer are associated with a decrease in antioxidant response contributing to increased reactive oxygen species (ROS) and consequent oxidative damage with malignant transformation. In this situation, supplementation with oral antioxidants might impede the pro-cancerous oxidant actions. During the progression of cancer, malignant cells enhance their metabolic activity to meet their increasing energetic needs, resulting in a higher generation of ROS. To subsist in such condition, cancerous cells activate signaling pathways to increase the intracellular pool of antioxidant systems and protect themselves against excessive oxidative damage. Consequently, in late stages of cancer, supplementation with antioxidants could actually strengthen tumor protection and help malignant growths avoid cell death

Based on the idea that exercise training acts as an antioxidant in tumors (Gueritat et al. 2014), exogenous antioxidants may induce similar effects and, therefore, could substitute physical exercise in advanced stages of cancer, when its use is not tolerated by the patient. Nutritional support, including antioxidant supplementation, is generally used to reduce the adverse side effects of anticancer therapies, like muscle wasting, mucositis, and fibrosis (Fig. 3) (Borek 2004).

However, recent preclinical and clinical findings showed antioxidant treatment for aggressive cancers was detrimental. The Swedish team of Martin Bergo demonstrated that vitamin E or N-acetylcysteine (NAC) reduced survival and accelerated lung cancer progression in mice (Sayin et al. 2014) and enhanced malignant melanoma metastasis to lymph nodes and lungs in mice with *Braf* and *Pten* mutations (Le Gal et al. 2015). Similarly, Piskounova et al. showed NAC increased the number of melanoma cells in blood and metastatic burden in NSG mice bearing aggressive melanoma xenografts from patients (Piskounova et al. 2015). Consistent with these findings, Assi and colleagues found that supplementation with an antioxidant mixture containing vitamins, oligoelements, and polyphenols reduced survival and enhanced C26 colon tumor growth in cachectic mice (Assi et al. 2016).

On the contrary, studies using a low grade cancer model, antioxidant supplementation prevents tumorigenesis. Indeed, Gueritat and colleagues observed that natural antioxidants reduced the volume and delayed the growth of low-grade AT-1 prostate adenocarcinoma in rats (Gueritat et al. 2014). Similar findings occurred in mice bearing differentiated breast MCF-7 tumor (Liao et al. 1995) or Dalton’s lymphoma ascites (Sharma and Vinayak 2013). Clinically, histological studies found a decrease in the expression/activity of antioxidant enzymes in precancerous tissues (Bostwick et al. 2000; Cullen et al. 2003; Sander et al. 2003). In contrast, advanced grades of cancer showed increased expression/activity of antioxidant enzymes in tumor tissues (Sander et al. 2003; Durak et al. 1993; Portakal et al. 2000; Strzelczyk et al. 2012). The combination of preclinical and clinical data let us suppose that interventions with antioxidants could be plausible at early stages of cancer, but harmful in advanced stages.

### Oxidatively generated DNA damage: potential biomarkers for cancer progression monitoring which need to be correctly assessed

The dose-dependent effects of physical exercise or antioxidant supplementation supports the idea that monitoring tumor redox status could be a promising therapeutic strategy in the care management of cancer patients. Among the different biomarkers that could be used to assess redox status of tumor cells, oxo-7,8-dihydro-2′-deoxyguanosine (8-oxodG) has gained much attention. However, its role as a reliable marker of cancer progression is still under debate, as its levels in blood, urine, or tumor tissues to predict survival and efficacy of anticancer treatment is still under debate (Sheridan et al. 2009; Crohns et al. 2009; Roszkowski et al. 2011; Roszkowski and Olinski 2012; Shen et al. 2007; Karihtala et al. 2011; Lee et al. 2010a).

The difficulties to establish links between cancer progression and levels of oxidatively generated DNA damage is largely explained by inaccurate quantification of 8-oxodG. It is now well documented that several assays, including gas chromatography-mass spectrometry (GC-MS) and [^32^P]-postlabeling methods, suffer from major flaws during sample preparation (Cadet et al. 2011, 2012), leading to overestimation of 8-oxodG. In addition, immunoassays, which are still widely and improperly applied in clinical studies, are inaccurate due to antibody cross-reactivity (Lee et al. 2010a; Cadet et al. 2011). High performance liquid chromatography coupled with electrospray ionization tandem mass spectrometry (HPLC-ESI-MS/MS) in the multiple reaction monitoring mode has become a relevant alternative thus providing unambiguous damage characterization and quantitative information (Lee et al. 2010a; Cadet et al. 2011). However assessing low variations in 8-oxodG levels by HPLC-ESI-MS/MS is still a challenge (Badouard et al. 2008). An additional alternative is the indirect detection of 8-oxodG or oxidized pyrimidine bases, mediated by either the comet assay or the alkaline elution technique, in association with DNA repair enzymes that convert base modifications into strand breaks (Klungland et al. 1999; Sauvaigo et al. 2002).

The measurement of 8-oxodG and 8-oxoGua, the related nucleobase, in human fluids including urine, plasma, and saliva offers a non-invasive method to indirectly assess oxidatively generated damage in nuclear DNA (Cooke et al. 2008). However, the biological meaning of urinary 8-oxodG remains to be established since it was recently reported that the release of the oxidized nucleoside could not be accounted for by either the nucleotide excision repair from DNA or MTH1-mediated hydrolytic dephosphorylation of 8-oxo-7,8-dihydro-2′-deoxyguanosine 5′-triphosphate in nucleotide pools (Evans et al. 2016).

These studies show that tumor redox status affects tumor development and impacts anticancer therapies. Physical exercise and antioxidant supplementation are proposed to optimize the effects of some specific agents, while being deleterious for Taxane family drugs. Future steps will require the identification of a patient’s tumor redox status. To achieve this goal requires the identification and validation of biological markers of oxidative stress to make the right diagnosis. As such, oxidatively DNA damage constitutes a promising biomarker, but requires an improved standardized and clinically relevant approach.

## Neurological disorders and stroke

### Stroke

There is a long-standing link between neurodegenerative disorders, stroke, and oxidative stress. In general, the brain is particularly sensitive to oxygen overload and free radical generation, as it requires higher levels of oxygen and glucose than do other organs and tissues. Additionally, the brain’s high amounts of unsaturated fatty acids, with their easily oxidized double bonds, makes it highly susceptible to free radical attack (Butterfield et al. 2002). In strokes and the onset of cerebral ischemia, a decrease in oxygen levels removes controls over the free radicals generated by electron transport and leads to decreased levels of antioxidants, such as vitamins E and C, as they are consumed in the attempt to quench the increase in free radicals (Flamm et al. 1978). Even following the immediate danger of a stroke, free radicals continue to exert detrimental effects that lead to other complications such as brain edema, which can result from disruption of the blood brain barrier leading to fluid accumulation (Heo et al. 2005). There are some promising results suggesting that free radical scavengers may be beneficial in remediating post-stroke symptoms in a clinical setting (Toyoda et al. 2004), making antioxidant therapies a potentially promising avenue for treatment following strokes. Another type of stroke associated with oxidative damage is intracerebral hemorrhage or hemorrhagic stroke. In this type of stroke, free radicals are produced in two ways, the first is through the decomposition of blood that has rapidly accumulated in the brain, which in turn releases iron ions, heme, and thrombin that induce the production of free radicals. Second, the inflammatory response by cells such as microglia and neutrophils also generate free radicals. Together both sources of free radicals are leading causes of hemorrhagic brain tissue (Duan et al. 2016). These examples illustrate that often the events following a stroke are just as lethal with oxidatively generated damage playing a key role in the toxicity, making antioxidant therapies a promising treatment option.

### Alzheimer disease

Neurological disorders such as Alzheimer’s disease, amyolotrophic lateral sclerosis (ALS), Multiple Sclerosis (MS), and Parkinson’s disease all include elements of redox imbalance (Uttara et al. 2009). Whether free radicals and oxidative stress are involved in both the initiation and the progression of (each of) these diseases, however, is not so clear. For example, Alzheimer disease is characterized by the accumulation of amyloid-*β* peptides to form amyloid plaques, which cause neuronal death (Butterfield et al. 2002). Evidence indicates that these aggregates then interact with redox metals to initiate the production of free radicals further promoting oxidative stress (Varadarajan et al. 2000). The inability to maintain a homeostatic balance between production of amyloid-*β* peptides and their degradation results in not only the elevation of oxidative stress but also the inhibition of proteasome-driven protein degradation (Bonet-Costa et al. 2016), which further contributes to the cascading effect of amyloid plaque formation. Exhibiting a similar neuropathology to Alzheimer disease is Down Syndrome (DS), a congenital birth defect that is the result of three copies of chromosome 21. Similar to Alzheimer disease, a redox imbalance likely has some role in the adverse effects of DS. One gene coded on chromosome 21, CuZn superoxide dismutase, has activity increased by 50% in DS. This enzyme is responsible for catalyzing the dismutation of superoxide to molecular oxygen and hydrogen peroxide, and increased activity of this enzyme may lead to greater production of hydrogen peroxide (Brugge et al. 1992). Additional similarity between DS and Alzheimer disease is found in the formation of amyloid plaques. In DS, the gene for amyloid A4 precursor is also located on chromosome 21 and is present at a rate 50% higher than normal (Rumble et al. 1989), which is potentially responsible for the amyloid plaque formed in DS. RCAN1, a regulator of calcineurin, has also been linked to both DS and Alzheimer disease as well as another neurological disorders. Paradoxically, RCAN1, specifically the isoform RCAN1-1L is neuroprotective against acute stress, but has detrimental effects when chronically up-regulated. RCAN1-1L is transiently elevated to defend against acute stress including oxidative stress; however, long-term elevation of RCAN1-1L is linked to DS and Alzheimer disease (Ermak and Davies 2013). In patients with Alzheimer disease, RCAN1-1L is expressed twice as much relative to non-Alzheimer disease controls suggesting that RCAN1-1L may have a role in Alzheimer disease (Ermak et al. 2001; Harris et al. 2007).

For the individual, environment factors are major determinants of health across the lifespan. Further studies of ApoE alleles on ageing processes need also to consider environmental influences because the heritability of human lifespan is 30% or less (Finch and Tanzi 1997; Dato et al. 2017). The “bad” ApoE4 allele is associated with shorter lifespan and higher risk of Alzheimer disease and cardiovascular disease. Its life-shortening association was first reported in 1994 by Schächter and colleagues at the *Centre d’Etudes du Polymorphisms Humain* (Schächter et al. 1994).

However, ApoE4 is protective in some environments with high exposure to pathogenic infections. In rural Ghana, ApoE4 had allele-dose benefits to survival of adults and children (van Exel et al. 2017). Moreover, the Tsimane of low-land Bolivia had better cognitive functions in ApoE4 carriers who also had blood indicators of a high parasite load (Trumble et al. 2017). Because ApoE4 is the most ancient allele (Fullerton et al. 2000), its persistence in human populations may represent its adaptive value under conditions of high pathogen exposure (Finch and Stanford 2004; Martin and Finch 2016). The lower risk of age-related macular degeneration (AMD) in ApoE4 carriers (Section 12.3) might be related to the increased UV exposure of early humans in the African savannah, where there is greater solar exposure than in the forest canopy of our great ape ancestors. UV-induced retinal damage is implicated in AMD (Kraljević Pavelić et al. 2015) (Section 10.4).

In our highly polluted world, ApoE4 may increase the risk of Alzheimer disease. Based on epidemiological findings from the Womens’ Health Initiative Mental Study (WHIMS), it was shown that older women residing in elevated particle density (PM2.5, above EPA standards) had nearly double risk of dementia, with further doubling of risk in E4/E4 carriers (Cacciottolo et al. 2017). Correspondingly, EFAD mice carrying transgenes for ApoE alleles and familial Alzheimer disease had greater bran amyloid in the E4FAD carriers. Given the low heritability of human lifespan, it is timely to expand environmental studies in discussions of human ageing mechanisms*.* The human environment is being re-conceptualized in terms of the *exposome*, to identify external and internal influences on individual health and their synergies across the lifespan (of, N.A. and E. Sciences, and Medicine 2017; Vineis et al. 2017). The search for longevity associated gene variants might also consider environmental factors that contribute to life-shortening diseases.

### Huntington disease

In contrast with high levels of RCAN1 expression in Alzheimer disease, depressed levels of RCAN1-1L are a hallmark of Huntington’s disease, a disease characterized by neuronal death caused by a mutant huntingtin protein (Ermak et al. 2009). RCAN1-1L has been shown to protect against toxic mutant huntingtin proteins in vitro by inhibiting calcineurin, which in turn increases phosphorylation of the mutant huntingtin protein. In Huntington disease, there are elevated levels of biomarkers for oxidative damage such as DNA damage and oxidized proteins, which is believed to be the cause of mitochondrial dysfunction and neuronal cell death. The association between oxidative stress and Huntington disease is well documented, and multiple studies and some clinical trials have shown antioxidant therapies to be a promising candidate for slowing progression and alleviating symptoms of Huntington disease (Gil-Mohapel et al. 2014).

### Multiple sclerosis

MS is an auto-immune neuronal disorder that causes demyelination of the central nervous system (CNS). Due to the abundance of reactive oxygen species, it is arguably the disease source; however, the exact source of MS is still unknown. Analysis of MS plaques revealed elevated reactive oxygen species generation and decreased antioxidant levels (Langemann et al. 1992). Even though a cure to MS has yet to be developed, dietary modifications have shown promising results in delaying and easing symptoms of MS. A low fat diet has been shown to result in lower deterioration and death rates (Swank and Dugan 1990). Similarly, a low fat diet supplemented with the omega-3 fatty acids from fish oil has also been shown to decrease the severity of MS symptoms (Weinstock-Guttman et al. 2005). These studies show that diets high in antioxidants and low in oxidative strain have the potential to alleviate symptoms of MS and delay its progression through the sequestration of free radicals and restoring redox balance.

### Parkinson disease

Parkinson disease is characterized by a neuronal loss, especially the nigral dopaminergic neurons and intraneuronal inclusions called Lewy bodies and constituted mainly of α-synuclein. This results in the underrepresentation of the neurotransmitter dopamine which is at origin of the cardinal motor symptoms of the disease: akinesia rigidity and tremor. The cause of neuronal loss is not fully known but includes aggregation of misfolded proteins (α-synuclein, tau), mitochondrial dysfunction (especially a loss of complex 1 activity), defective ubiquitine–proteasome proteolysis, and oxidative stress.

Ultimately, dopamine neurons degenerate by an apoptotic process involving caspase 3 and 8 activation (Hartmann et al. 2000; Hartmann et al. 2001). In addition to these so-called cell autonomous mechanisms, non-cell autonomous mechanisms such as neuroinflammatory processes or dysregulation of trophic support also contribute to neuronal degeneration and self-perpetuation of degeneration (for review see (Hirsch and Hunot 2009)). This includes activation of microglial cells secreting pro-inflammatory cytokines and infiltration of CD4 and CD8 T lymphocytes as evidenced post-mortem in the substantia nigra of patients with Parkinson disease (McGeer et al. 1988; Brochard et al. 2009). Experimental models, mainly developed in mice using the parkinsonian drug MPTP, suggest the activation of both innate and adaptive immune response is involved in neuronal degeneration as blockade of these events prevents at least partially the loss of dopamine neurons.

Several lines of evidence suggest oxidative stress plays a very special role in the degeneration of melanized dopamine in Parkinson disease, which are particularly sensitive to oxygen radicals (Hirsch et al. 1988). Within dopaminergic neurons, oxidative stress is due in part to a deficiency of complex-1 activity within mitochondria (Schapira et al. 1990). In line with this hypothesis, complex-1 inhibitors such as MPTP, rotenone, and the recently discovered annonacin can killed dopaminergic neurons in vitro and reproduce parkinsonism in animals (Lannuzel et al. 2008; Champy et al. 2004; Höglinger et al. 2003). Rotenone and annonacin are of great interest as they also produce α-synuclein and tau accumulations within the degenerating neurons. Post mortem analysis confirms that oxidative stress participates in neuronal degeneration through increased lipid peroxidation (Dexter et al. 1986), protein carbonylation (Alam et al. 1997a), and 8-hydroxyguanine formation (Alam et al. 1997b), all of which have been reported in the parkinsonian substantia nigra. Furthermore, oxidative stress is amplified by a rise in iron concentration (Hirsch et al. 1988; Sofic et al. 1988; Dexter et al. 1987). The origin of this increase in nigral iron is not fully understood but might be due to a dysregulation of iron transport within the cells. Indeed, the divalent metal transporter (DMT1) is over-expressed in Parkinson disease and animals with a mutated DMT1 (reducing its iron transport capacities) were less sensitive to parkinsonian toxins (Salazar et al. 2008). Altogether, multiple sources of evidence suggest oxidative stress, involving a rise in iron and the subsequent accumulation of misfolded proteins within dopaminergic neurons participate in the degenerative process. This raises the tantalizing hypothesis that reducing iron concentration in Parkinson disease might reduce the evolution of the disease. A recent clinical trial in France using the iron chelator deferiprone actually provided encouraging support for this hypothesis (Devos et al. 2014) which will be further tested (and hopefully confirmed) in an ongoing European clinical trial.

There is also the possibility that diet and antioxidants may affect the incidence or progression of Parkinson disease. In Parkinson disease, protein damage and degradation mediated by reactive oxygen species and a positive feedback loop (Esteves et al. 2009) appear to contribute significantly to the loss of dopaminergic cells in the substantia nigra, leading to dopaminergic deficiency in the striatum, and alpha-synuclein oligomerization. Conversely, administration of antioxidants (GSH and coenzyme Q) decreased alpha-synuclein oligomerization and thus neurotoxicity (Esteves et al. 2009). Several studies have shown certain diets exhibited neuroprotective patterns against Parkinson disease. One study showed an inverse relationship with Parkinson disease and adherence to a Mediterranean-type diet (Alcalay et al. 2012), while other studies showed that high consumption rates of plant-based diet (such as fruits, vegetables, legumes, whole grains, nuts) with fish and poultry was inversely associated with Parkinson disease risk when compared with a standard Western diet characterized with more red meat, sweets, and fried foods (Gao et al. 2007). Similar to other neurological disorders covered, diet can play a large role in improving quality of life in patients with Parkinson disease and offer a promising avenue of further study.

#### Exercise enhances neuroplasticity by targeting synaptic energy metabolism and mitochondrial function in Parkinson disease patients

Despite the brain’s high metabolic demand, it has little reserve when substrates are limited. Failure to supply neurons with energy can be severe, leading to neurotransmitter dysfunction and cell death. The analyses of brain tissues from a wide spectrum of neurodegenerative disorders all implicate deficits in the mitochondria. Consequently, mitochondria-targeted pharmacological therapeutics have been tested. However, none have translated into successful clinical treatments. Despite these challenges, energy metabolism remains a viable therapeutic target. By better understanding the underlying molecular and biochemical changes in glucose metabolism within the brain, including conditions of metabolic stress, such as transient hypoxia, acute and chronic injury, and degenerative diseases, may provide better direction of targeted therapies.

Over the past several years, exercise has become attractive as a non-pharmacological and inexpensive treatment modality for many brain disorders. For example, many Parkinson disease patients incorporate aspects of physical activity in their standard of care. Small studies and patient reports support the benefits of exercise in improving quality of life, optimizing drug treatment, and possibly modifying disease progression. Research in animal models highlight the beneficial effects of exercise, and further our understanding of the underlying molecular mechanisms. Even in light of these major advancements, major gaps still exist.

The following studies highlight the hypothesis that physical activity in the form of different types of exercise (aerobic versus skill-based) is beneficial due to its circuit specific effects on synaptic connectivity (a major component of neuroplasticity). Specifically, the effects of exercise on energy metabolism at the synapse, its ability to modulate synaptic mitochondrial function, glucose uptake, astrocyte-neuron coupling, and the mechanisms that link central nervous system (CNS) and peripheral (muscle) activity. Together, a greater understanding of these processes may improve pharmacological drug development, guide clinicians on patient-appropriate exercise, especially in treating motor deficits associated with Parkinson disease, and provide insight on long-term exercise for optimal brain function, including the later stages of ageing.

#### Effects of exercise in an animal model of Parkinson’s disease

Epidemiological studies have shown that exercise and other forms of physical activity reduce the incidence of Parkinson disease, Alzheimer disease, and mild cognitive impairment (MCI) (Chen et al. 2005; Ohman et al. 2016). Animal models utilizing the dopamine depleting agents MPTP (1-methyl-4-phenyl-1,2,3,6-tetrahydropyridine) and 6-OHDA (6-hydroxydopamine) have been instrumental in our understanding.

However, a limitation of these studies is lack of consensus on exercise parameters employed in mouse studies, including duration, velocity, intensity, and level of skill. However, one common component is exercise introduced prior to or during the active phase of toxin-induced cell death protects against midbrain dopaminergic neuron cell death and the depletion of striatal dopamine. A number of mechanisms may mediate neuroprotection including (i) elevation of neurotrophic factors such as BDNF (brain-derived neurotrophic factor), (ii) increased resilience of mitochondria, (iii) reduced bioavailability of toxins due to alterations in peripheral processing (liver metabolism), transport across the blood brain barrier, uptake into dopaminergic neurons (through reduced expression of the dopamine transporter, DAT), or storage/sequestration via vesicular uptake through VMAT2 (vesicular monoamine transporter) into vesicles.

Dopaminergic toxin-induced models can also be used to study neurorestoration: the effects of exercise on neuronal connectivity following toxin-induced cell death. One area of interest is understanding the underlying mechanism by which exercise, in the form of intensive treadmill running, leads to the restoration of motor behaviors in the MPTP-lesioned mouse model, collectively termed neuroplasticity (Fisher et al. 2004). In summary, exercise led to elevated striatal dopamine D2 receptor expression, reduced striatal DAT expression, elevated release of dorsolateral striatal dopamine, altered expression of the ionotropic glutamate receptor subunits GluA1 and GluA2, and restoration of dendritic spine density on striatal medium spiny neurons (MSNs) (Fisher et al. 2004; Petzinger et al. 2007; Toy et al. 2014; Kintz et al. 2013; VanLeeuwen et al. 2010). These results indicate circuit specific changes leading to improved motor behavior. The concept of circuit-specific changes is further supported by recent studies examining alterations in regional cerebral blood flow (rCBF) with exercise. Specifically, elevation in cortical rCBF in the PFC, with enhanced distribution upon skilled exercise compared to aerobic exercise (Wang et al. 2015).

While exercise provides general brain health, we are interested in understanding how exercise can be targeted to specific brain circuits, especially those affected in neurological disorders like Parkinson disease. Moreover, different types of activity have variable effects. For example, types of exercise with greater skill and learning components, which engage cognitive circuits, show greater benefit than those which are purely aerobic and limited cognitive engagement (Voelcker-Rehage et al. 2011). This is important in patients with Parkinson disease where cognitive deficits are evident in the early stages of disease and may contribute to the degenerative process and lead to the progression of severe motor deficits. Interestingly, early synaptic changes with exercise in the MPTP-lesioned mouse model originate from cortical afferents, especially the corticostriatal circuit connecting the cerebral cortex with the striatum (caudate-putamen). These observations, along with the fact that deficits in cognition can interfere with gains in the benefits of exercise, highlight the importance of circuit specific targets that must be engaged to enhance improvements in motor behavior. In other words, mechanisms of neuroplasticity enhanced by activating cognitive circuits are critical for targeting improvement in motor circuits.

Therefore, a major gap in our knowledge is the identity of the molecular mechanisms by which exercise, especially forms of exercise targeting corticostriatal afferents, lead to improved motor behaviors and modify disease progression. One attractive hypothesis is the role of metabolism as it links neuronal activity-induced changes in blood flow, breakdown of energetic substrates to the expression of genes and proteins involved in angiogenesis and synaptogenesis. Central to this hypothesis are the biosynthetic mechanisms involved in energy metabolism, mitochondrial function, and synaptic connectivity.

#### Brain energy metabolism in Parkinson’s disease

The brain is the most metabolically demanding organ in the human body (Attwell and Laughlin 2001). However, unlike most peripheral tissues, the brain has limited metabolic flexibility such that it relies almost exclusively on glucose (and under specific conditions, lactate) as its substrate for energy metabolism. This highlights the brain’s dependency on a continuously available exogenous fuel supply. In the brain, glucose is metabolized through four principle biochemical pathways: (i) glycolysis, (ii) oxidative phosphorylation (OXPHOS), (iii) the pentose phosphate pathway (PPP), and (iv) glycogenesis. Importantly, each of the different cell types within the brain (neurons, astrocytes, microglia, oligodendrocytes) has its own distinctive metabolic profile. For example, neurons have a high energy expenditure, due to the establishment and maintenance of the membrane resting potential. In addition, neuronal computation is extremely energetically demanding, with most energy consumption occurring at the synapse (Harris et al. 2012). This demand is reflected by the high number of mitochondria preferentially localized to the pre- and post-synaptic terminals, implicating the close relationship between energy production and its demand (Wong-Riley 1989). Mitochondria not only produce ATP, but are involved in other molecular functions, including buffering the cellular calcium influx and the generation of low levels of reactive oxygen species necessary for normal metabolic homeostasis.

Loss of mitochondrial function at synapses can lead to synaptic deregulation and loss of brain connectivity. Historically, the study of neurodegenerative disorders has correlated alterations of mitochondrial function with synaptic changes. With respect to Parkinson disease, the discovery of MPTP-induced parkinsonism shed light on mitochondria as a contributor to disease process (Langston et al. 1983; Langston 2017). Since then, the involvement of mitochondria has been shown to be much more complex, with defect in: trafficking, biogenesis, mitophagy, activation of apoptosis, oxidative stress, and calcium buffering capacity (Mounsey and Teismann 2011).

In addition, the mechanisms by which familial forms of Parkinson disease occur have increased our insight. These familial forms of Parkinson disease include genetic mutations in alpha-synuclein (SNCA), DJ-1, PINK1, Parkin, LRRK2, and UCH-L1. Many of these genes play key regulatory roles in mitochondrial function and synaptic health, especially those localized to the nigrostriatal and corticostriatal pathways. The PINK1/Parkin pathway acts as a mitochondrial “quality-control” pathway, due to its role in Drp1-mediated mitochondrial fission. Mutations in this genetic pathway are implicated in Parkinson disease characteristic dopaminergic degeneration (McWilliams and Muqit 2017). Similarly, loss of DJ-1 causes accumulation of dysfunctional mitochondria at the synapse by disrupting lysosomal homeostasis (Krebiehl et al. 2010). LRRK2 mutations act as an underlying cause of mtDNA damage in neural cells, the downstream effects impacting calcium imbalance, oxidative stress, and dendritic shortening. Alpha-synuclein impairs normal mitochondrial fission (biogenesis) (Xie and Chung 2012).

Exercise is inherently associated with metabolism since increased metabolic expenditure demands require increased ATP. At the neuron, energy demands rise with increased firing of action potentials and increased synaptic neurotransmission. These metabolic challenges are addressed by enhanced delivery of glucose and lactate to meet the demands for greater ATP production. In addition, neuroprotective strategies are also activated through metabolic responses that help to protect neurons from acute bouts of oxidative stress; mitochondria can enter a state of senescence while alternative sources of energy production are activated—including aerobic glycolysis for the production of ATP (Barros 2013).

In addition to increased energy requirements, the brain also engages in anabolic processes by which structural proteins and other macromolecules necessary for neuroplasticity including the synthesis of nascent receptors, axonal and dendritic scaffolding proteins, and other morphological changes are produced. The metabolic pathways, either directly or indirectly, contribute in shuttling small molecular substrates such as glucose, lactate, and other molecules like lipids and amino acids in supplying the needed structural components for neuroplasticity. Evidence is now emerging that exercise, specifically forms of exercise that target corticostriatal circuits, can promote metabolic changes that can harness neuroplasticity.

Exercise activates specific circuits in the brain, including those involved in motor control, linking the cerebral cortex, basal ganglia, thalamus, and cerebellum. Enhanced neuronal activity is associated with increased metabolic demand and elevated levels of local cerebral glucose utilization. Consistent with this, several groups have reported increased expression of the glucose transporters GLUT1 (endothelial- and astrocyte-specific) and GLUT3 (neuron-specific) in motor control regions following treadmill exercise (Allen and Messier 2013; Takimoto and Hamada 2014; Kinni et al. 2011). Although there have been no reported changes in GLUT1 expression in the Parkinson disease brain, it has been shown that mitochondrial dysfunction and reactive oxygen species accumulation can impair cellular glucose uptake and an inadequate glucose supply can have deleterious effects on dendritic morphology and impair synaptic plasticity, which underlie aspects of Parkinson disease pathology (Gandhi et al. 2009). Therefore, by driving activation of circuits affected in Parkinson disease, exercise imposes a metabolic challenge, which is met by an adaptive upregulation of glucose transporters to improve glucose availability and to support synaptic function. In addition to changes in GLUT1 expression, there is also an activation of enzymes involved in energy production including dehydrogenases for lactate and glucose and others within the TCA cycle.

In recent decades, lactate has emerged from largely being considered as a metabolic waste product to an important supplemental brain energy substrate and signaling molecule. At rest, blood-derived lactate (~ 1 mM) provides 8–10% of total brain energy requirements (Smith et al. 2003; Boumezbeur et al. 2010). Yet, during moderate-to-vigorous aerobic exercise, blood lactate levels can increase by up to 10-fold, supplementing brain energy requirements, up to 25% of the overall demand (van Hall et al. 2009). Complementing elevated blood lactate levels following bouts of aerobic exercise, specific regional up-regulation of lactate transporters have been found in the cortex, hippocampus, and hypothalamus, indicating a preferential utilization of peripheral lactate during supra-physiological conditions (Takimoto and Hamada 2014). Furthermore, it has been suggested that the immediate energy requirements of sustained synaptic activity are at least, in part, fueled by the complete oxidation of astrocyte-derived lactate (Hall et al. 2012). The potential central role of astrocytes in linking metabolism and neuroplasticity is highlighted in the next sections.

#### Effects of exercise on brain mitochondrial function in Parkinson disease

It has been well-established that aerobic exercise training is highly beneficial to muscle mitochondria efficiency. Surprisingly, much less is known about the effects of exercise on mitochondria in the brain, either in neurons or glia. As mentioned above, neuronal mitochondria are preferentially localized to the synapse and are responsible for supplying the energy demands of synaptic function in active neuronal circuits. In normal ageing and in neurodegenerative disorders such as Parkinson disease, there are a number of mitochondrial alterations that can result in their dysfunction, including the following: decreased activity of the ETC and other enzymatic proteins, including those involved in controlling oxidative stress and calcium buffering, accumulation of mtDNA mutations, altered biogenesis, and dysregulation of mitophagy (Schapira et al. 1990). Exercise represents a potentially important non-pharmacological approach to target mitochondrial function at the synapse with the goal to facilitate synaptic integrity and to promote synaptic plasticity.

One means by which exercise can modulate brain mitochondria is through improved respiratory chain function. For example, regular physical activity has been reported to increase enzymatic activity of ETC Complexes I, III, and IV (Navarro et al. 2004). Furthermore, improved motor behaviors following exercise in the MPTP mouse model of Parkinson disease is associated with enhanced mitochondrial function (Lau et al. 2011). In addition to affecting mitochondrial ATP production efficiency, exercise can also regulate mitochondrial density at the synapse. Chronic physical exercise training, such as treadmill running, up-regulates the genes for silent information regulator 1 (SIRT1) and peroxisome proliferator-activated receptor-γ coactivator 1-alpha (PGC-1α). These proteins regulate mtDNA copy number in several brain regions including the PFC, striatum, and hippocampus, as well as cascades of enzymes involved in energy metabolism, biogenesis, regulation of oxidative stress, mitophagy, and apoptosis (Steiner et al. 2011; Gerhart-Hines et al. 2007). It is of interest to note that SIRT1 and PGC-1α are the focus of a number of pharmacological agents to enhance mitochondrial health and one can speculate that efficacy of any drug intervention may be enhanced with the synergistic application of exercise.

#### Astrocytes, exercise, and neuroplasticity in Parkinson disease

Astrocytes fulfill important tasks, including mediating brain vasculature, providing energetic substrates (such as lactate) for neighboring neurons, and recycling synaptic glutamate following neuronal activity. The latter activity, the glutamate-glutamine cycle, is a cornerstone of astrocyte-neuron coupling and serves as a key mediator of neuronal activation. With astrocytic processes enveloping neuronal synapses, the astrocytic excitatory amino acid transporter 1 and 2 (EAAT1 and 2) serve as sensors of neuronal activation (and subsequent glutamate release) and facilitate uptake of glutamate from the synaptic cleft (Rothstein et al. 1994). This glutamate is then converted into glutamine, which is subsequently recycled back to neurons for conversion into glutamate and repackaged into vesicles for future synaptic events. The uptake of glutamate is an ATP-dependent process, due to increased sodium-potassium-ATPase activity to meet ionic displacement during glutamate uptake (Khatri and Man 2013), thus inducing shifts in astrocytic metabolism to meet the neuroenergetics demands of glutamate-glutamine cycling.

This example of astrocyte-neuron coupling forms the basis for the astrocyte-neuron lactate shuttle (ANLS) hypothesis which postulates that (i) excitatory neurotransmission at the glutamatergic synapse (glutamate/glutamine cycle) drives glucose uptake from the circulation via GLUT1 expressed in microvascular endothelial cells and astrocytes, (ii) glucose is preferentially metabolized via aerobic glycolysis in astrocytes to yield lactate, and (iii) lactate is shuttled from astrocytes to neurons via monocarboxylate transporters (MCTs) where it is converted back to pyruvate and enters the TCA cycle in mitochondria (Pellerin and Magistretti 1994). Astrocyte-specific metabolic changes during high levels of neuronal activity serve not only to provide energy to excitatory neurons through the ANLS, but also a necessary metabolic pivot to continue cycling glutamate.

Important for the facilitation of exercise-induced neuroplasticity, the ANLS represents a model, which couples neuronal activity and the rapid delivery of energy substrates to the synapse. Preliminary data suggest that exercise may target synaptic metabolism by up-regulating the expression of ANLS components, such as lactate dehydrogenase and MCT2 (neuron specific lactate transporter), in the striatum, following a single bout of treadmill exercise (unpublished data, Halliday et al.). Interestingly, cell-specific overexpression of GLUT1 (in astrocytes) and MCT2 (in neurons) has been shown to be protective against glutamate excitotoxicity. Taken together, these findings suggest that exercise may improve astrocyte-neuron cooperation to facilitate lactate delivery required by active synapses in order to supplement the increased metabolic demand, and thus support synaptic function and plasticity. Disruption of astrocytic lactate impairs long-term memory formation and is rescued by administration of exogenous lactate, but not glucose (Suzuki et al. 2011). Intra-hippocampal infusions of lactate enhance working memory and blocking neuronal uptake of lactate impairs memory formation in rats (Newman et al. 2011). Further, neuronal uptake of lactate induces activation of immediate early genes (IEGs), including Zif-268, c-Fos, and Arc, implicating lactate in neuroplasticity processes (Yang et al. 2014). These findings, and others, demonstrate the critical role of astrocyte-derived lactate serving as an important energy substrate in synaptic plasticity.

The close relationship between astrocytes and neurons and their health is paramount to adaptive neuroplasticity and synaptogenesis. In a number of neurodegenerative disorders, astrocytic activation has been well documented in terms of astrocyte morphology and elevated glia fibrillary acidic protein (GFAP) expression, a marker of astrocyte inflammation. Indeed, neuronal expression of lactate dehydrogenase subunit 1 (LDH-1) demonstrates a cell-specific ability to oxidize lactate (Bittar et al. 1996). An outcome that may prove beneficial in supplying high energetic demands during oxidative stress when glucose is selectively shunted toward the PPP to maintain neuronal antioxidant status. Additionally, overexpression of lactate dehydrogenase in vitro provided protection against amyloid-β exposure by shifting neuronal energetics from OXPHOS to aerobic glycolysis, ensuring cellular resistance (Newington et al. 2012). Dysfunctional astrocytes can have extensive molecular consequences including reduced lactate production to supply neurons, dysfunction of the BBB at astrocyte end feet, and diminished support through factors such as BDNF. Thus, astrocytic integrity may serve as an early indicator of brain disease and may emerge as an important therapeutic target.

#### A potential role for hypoxia-inducible factor 1 in Parkinson disease

When local metabolic demand through elevated neuronal activity exceeds that of available energy resources, highly conserved transcriptional programs are activated to increase oxygen delivery, optimize cellular metabolism, increase metabolic capacity, and establish homeostasis. The wide spectrum of molecular targets necessary for a successful response indicates the importance and complexity of changes that are necessary to survive challenges like metabolic demand. One such candidate is the hypoxia-inducible factor-1α (HIF-1α), a member of the HIF family of proteins. HIF-1α is a cytoplasmic multimeric protein that is constitutively expressed. Under normoxic conditions, HIF-1α is hydroxylated and subsequently ubiquitinated for targeted proteasome degradation. Under conditions of low oxygen, such as the transient hypoxia state during intensive exercise, HIF-1α is stabilized, and translocates into the nucleus, and in complex with the protein HIF-1β, forms an active transcription factor that binds to the hypoxia response element (HRE) found in the promoter region of a large number of genes including many responsible for glucose and lactate transport, energy metabolism, angiogenesis, mitochondria function, and synaptogenesis.

**Fig. 4:**
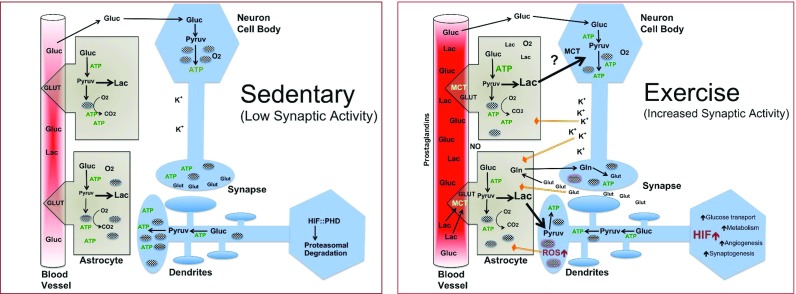
Changes in metabolism involving the corticostriatal pathway lead to improved circuit specific changes. The *left panel* highlights some of the features of the corticostriatal circuit in sedentary animals when synaptic activity is low. Glucose serves as the primary energy substrate in neurons catabolized to pyruvate by glycolysis and shuttled into mitochondria to the TCA cycle and ETC for oxidative phosphorylation. Mitochondria (small checkered ovals) generate ATP via oxidative phosphorylation. Expression of regulatory factors such as HIF is attenuated under normoxia. The *right panel* highlights changes in the corticostriatal circuit with exercise. Neuronal activation leads to significant metabolic changes at the synapse including reduced glucose and depletion of oxygen. Activation of astrocytes leads to an increase in aerobic glycolysis elevating levels of lactate from glucose substrates. Lactate is transported to neurons via the astrocyte neuron lactate shuttle (ANLS) for conversion to pyruvate providing an alternative substrate source for mitochondria energetics. Astrocytes located near synapses may have a unique function in neuroplasticity in that they can detect increased activity through changes in potassium release, glutamate neurotransmission, increased oxidative stress through ROS, and depletion of oxygen and glucose. It has yet to be established how these mechanisms may or may not differ from those astrocytes metabolically associated with neuron cell bodies for example. Changes in blood flow are both acute due to dilation by nitric oxide and prostaglandins and chronic due to increased expression of factors such as HIF-1α dependent VEGF. Increased blood flow and peripheral effects of exercise elevate blood glucose and lactate levels that can be transported to astrocytes and neurons via transporters including MCTs and GluTs

It has been suggested that one of the potential benefits of exercise in the MPTP-lesioned mouse model is through the activation of HIF-1α (Smeyne et al. 2015). Thus, the role of HIF-1α in neural tissues is not restricted to the regulation of energy metabolism through oxygen sensing but has a potentially wider role in regulating complex molecular events including neurogenesis, angiogenesis, and mitochondrial function (biogenesis), which are important for exercise-induced neuroplasticity and synaptogenesis (Zhu et al. 2005; Shweiki et al. 1992; Correia et al. 2011; Petzinger et al. 2015). Therefore, one potential mechanism may be that exercise, through increased cellular metabolic energy demand, activates the HIF-1α transcriptional program, which in turn supports synaptic strength and connectivity during motor learning in the Parkinson disease brain (Fig. 4).

It is interesting to note that transcription factors such as HIF-1α, which is tightly regulated by oxygen demand, are also regulated by other factors that can “sense” metabolic demand in neuronal circuits. These other factors include reactive oxygen species including superoxide, the glutamate-glutamine cycle, nitric oxide, and potassium levels all highlighting the potentially complex and important role played by such central regulators.

It is well-established that exercise is beneficial in both treating and preventing many disorders in the diseased and ageing brain. It is necessary to identify the underlying molecular mechanism such that novel treatments can target specific disorders. In terms of Parkinson disease, evidence indicates that circuit specific pathways linking cognitive and motor circuits are critical in enhancing neuroplasticity to generate benefits in complex behavior like movement. These studies highlight the potential link between energy metabolism and neuroplasticity. Circuit specific activation of neuronal pathways leads to changes in glucose and lactate metabolism, altered mitochondria function, and activation of cascades including those through regulators like HIF-1α that can induce genes and proteins necessary for angiogenesis, synaptogenesis, and other components of neuroplasticity. While this may not represent a cure for disorders like Parkinson disease, it does implicate an important therapeutic target to complement pharmacological strategies and if implemented early may in fact modify disease progression.

## Joints and inflammation

### Joints and mobility

Freely movable diarthrosis or synovial joints have the greatest range of motion of any joint in the body. The six types of synovial joints are the pivot, hinge, saddle, planar, condyloid, and ball-and-socket joints. Pivot joints include the atlantoaxial joint between the atlas and axis of the neck. Hinge joints are found in the interphalangeal joints of fingers and toes. Saddle joints occur between the trapezium and metacarpal I–thumb joint. Planar joints are found between the navicular and second cuniform of the ankle. Condyloid (or gliding) joints occur between the radius, scaphoid, and lunate bones of the wrist. Ball-and-socket joints are found in the hips and shoulders.

When diarthrosis or synovial joints are healthy, movement is smooth and painless. In contrast, when joints become inflamed and suffer damage to key components, movement becomes difficult and painful. Some common diseases or conditions that involve joint pain and inflammation are rheumatoid arthritis, systemic lupus erythematosus, and osteoarthritis, which are discussed below.

### Rheumatoid arthritis.

Rheumatoid arthritis was first described in 1800. Initially it was felt to be a variant of gout, but this idea was proven wrong when excess uric acid was found to be involved in gout, but not in rheumatoid arthritis (Garrod 1859). Various theories have been postulated on the etiology of rheumatoid arthritis from pathogens including bacteria, as well as various viruses as potential triggers (Fox 2005); however, a direct link has yet to be determined. A genetic component clearly plays a role as there is a clear association between a group of major histocompatibility complex cell surface receptors and disease predilection (Reveille 1998). An increased level of reactive oxygen species formation and oxidative stress has also been found in rheumatoid arthritis. Intracellular formation of reactive oxygen species, lipid peroxidation, protein oxidation, and DNA damage have all been found in the blood of rheumatoid arthritis patients (Mateen et al. 2016; Chuang et al. 2012), so there is clear evidence that oxidative stress occurs in rheumatoid arthritis patients. Lunec et al. (Lunec et al. 1985) proposed that oxidative damage to IgG generated protein aggregates that can activate neutrophils and set up a “vicious cycle” (Halliwell 1995) of free radical production. A more recent clinical target to attempt to control rheumatoid arthritis is tumor necrosis factor (TNF). Kageyama et al. (Kageyama et al. 2008) have reported that treatment with infliximab, an anti-TNF biologic, played an essential role as an antioxidative agent against advanced glycation end-product (AGE) formation, oxidative DNA damage, and lipid peroxidation.

### Systemic lupus erythematosus

Systemic lupus erythematosus is a complex inflammatory autoimmune disease whose etiology remains largely unknown. It involves many organs and presents in a varied way, mimicking many other diseases including malignancies and infections. Lupus typically presents with painful, swollen joints so that the disease is sometimes confused with arthritis. Lupus-related joint pain and stiffness are typically worse in the morning. In early stages of systemic lupus erythematosus, joint pain is typically mild, but then increases significantly as the disease progresses (Lahita 2011). The hallmark of systemic lupus erythematosus is the presence of diverse autoantibodies that are directed at a varied group of self-antigens (Boyd 1997). Many mechanisms have been implicated in its origin and disease progression. One of the mechanisms implicated is oxidative stress and free radicals. Uncontrolled oxidative stress is felt to contribute to modifications of cellular protein, lipid, and DNA. Biomarkers have been identified that may potentially be useful in measuring disease activity, and whose control could be an approach to diminish end-organ damage (Shah et al. 2014). Oxidative stress, for example, leads to lipid peroxidation these products have been implicated in the pathogenesis of systemic lupus erythematosus (Otaki et al. 2010). Autoantibodies are typically present several years prior to disease activity in systemic lupus erythematosus. Inflammation, infection, drugs, and environmental factors induce formation of neoantigens with involvement of reactive oxygen species. An attempt to determine how these factors trigger disease, in a subset of the population, is still under scrutiny. What is clear though is that oxidative damage is involved in diseases such as systemic lupus erythematosus. Formation of reactive oxygen species along with enzymatic and nonenzymatic control of these potentially harmful molecules becomes an ongoing process leading to disease (Kurien et al. 2012). Antioxidant enzymes may be the target of antibodies resulting in a loss of a delicate balance.

### Osteoarthritis

Osteoarthritis (OA) is a major cause of severe joint pain, disability, and diminished quality of life in ageing populations. Breakdown of the extracellular matrix of the articular cartilage is a major factor in development and progression of osteoarthritis. The exact mechanisms leading to this cartilage change have not been defined, but loss of glucosamine and hyaluronic acid are early findings, which may be genetically determined. Other early observations include cell loss, elevated oxidative stress, and increased expression of inflammatory mediators. Chronic age-related conditions such as osteoarthritis demonstrate de-regulation of common molecular and cellular mechanisms (Ladislas 2000). Some of these changes involve impaired autophagy, diminished clearance of apoptotic bodies, protein misfolding, and oxidative stress which (in turn) can activate pro-inflammatory pathways that are felt to contribute to degeneration of articular cartilage. Elevated serum levels of pro-inflammatory cytokines such as IL-1, IL-6, IL-18, TNFα, and CRP have been shown to increase with ageing (Krabbe et al. 2004; Cesari et al. 2004). Elevated levels of damaged proteins, a result of the increased oxidative stress, have been found in synovial fluid and plasma of osteoarthritis patients, and have been used as biomarkers for disease progression (Ahmed et al. 2016). The articular cartilage is present in joints to support and distribute mechanical loads, allowing motion while avoiding erosion. Most of the articular cartilage consists of thick extracellular matrix, whereas chondrocytes, the only cell type in the articular cartilage, represent only 5% of the joint mass. Ageing results in cell senescence (Musumeci et al. 2015), loss of chondrocyte phenotype and thus abnormal remodeling of the extracellular matrix (Quintero et al. 1984; Lotz et al. 2010), and age-related changes in the extracellular matrix (Bhosale and Richardson 2008). The interaction of all of these changes is still poorly understood and further investigation needed to determine age-related therapeutic targets that may eventually lead to treatment and/or prevention of osteoarthritis in the elderly.

## Bone and macrophages

Bone is a dynamic structure dependent upon the constant activities of osteoblastic bone formation and osteoclastic bone resorption. If these two actions are not in balance, pathology ensues (Boyle et al. 2003; Nohl 1993; Wauquier et al. 2009; Vaananen et al. 2000). There is growing evidence indicating increased reactive oxygen species-derived oxidative stress, which increases with the onset of an inflammatory state or with ageing, can adversely affect bone (Wauquier et al. 2009; Altindag et al. 2008; Garrett et al. 1990; Ozgocmen et al. 2007). Abnormally high levels of oxidants and/or low levels or activity of antioxidants can specifically impair the osteoblastic/osteoclastic balance. Under physiological conditions, cells derived from bone marrow macrophages, coined osteoclasts, produce oxidant species that facilitate the destruction of calcified tissue, thus participating in bone remodeling (Silverton et al. 1995; Yang et al. 1998).

Understanding which factors drive osteoclast activity and differentiation is important when studying diseases characterized by heightened bone resorption relative to formation, such as osteoporosis. In the last decade, studies have indicated that reactive oxygen species, including superoxide and hydrogen peroxide, are crucial components that regulate the differentiation process of osteoclasts.

### Oxidative stress and osteoporosis

A normal balance between oxidants and antioxidants is necessary for the correct equilibrium between osteoblast and osteoclast activities, respectively (Sheweita and Khoshhal 2007). In particular, when the production of reactive oxygen species by osteoclasts overwhelms the natural antioxidants defense mechanisms, the concomitant oxidant stress may lead to bone loss and hence to osteoporosis (Sheweita and Khoshhal 2007). However, the exact biochemical and cytological basis of the events finally responsible for bone resorption are still debated. It has been suggested that an enhanced osteoclastic activity may increase the superoxide anion generation and/or inhibit superoxide dismutase (SOD) and glutathione peroxidase activities with concomitant bone destruction (Sheweita and Khoshhal 2007). Indeed, early evidence for reactive oxygen species participation in bone reabsorption has been described (Garrett et al. 1990), wherein the addition of the superoxide-producing enzyme, xanthine oxidase, in vivo and in vitro, resulted in increased osteoclast numbers and activity. This effect was attenuated when SOD was added; however, no change was seen upon the addition of catalase, which scavenges H_2_O_2_. Therefore, the authors concluded that the superoxide radical rather than H_2_O_2_ or ·OH was important in mediating the enhanced bone resorption. Subsequent studies also found that O_2_^·–^ production was present within the osteoclast and suggested that it may be derived from nicotinamide adenine dinucleotide phosphate (NADPH) oxidase localized to the bone–osteoclast interface within the osteoclast ruffled border (Key et al. 1994; Steinbeck et al. 1994). Blockage of NADPH oxidase by diphenylene iodonium resulted in decreased O_2_^·–^ production and bone resorption (Yang et al. 1998; Darden et al. 1996). Other studies, however, indicated that rather than O_2_^·–^, H_2_O_2_ was the primary culprit behind osteoclast formation and activity (Bax et al. 1992; Fraser et al. 1996; Kim et al. 2006).

In a published case control study on 94 subjects aged > 60 years (50 healthy and 44 with osteoporosis), where total antioxidant status, plasma lipid peroxides, SOD, and glutathione peroxidase (GPx) activities were measured, oxidative stress appeared as an independent risk factor for osteoporosis linked to the increase of SOD/GPx ratio (Sanchez-Rodriguez et al. 2007). Furthermore, since increased osteoclastic activity and decreased osteoblastic activity were shown to be associated with an imbalance between oxidant and antioxidant status plasma biomarkers in postmenopausal osteoporosis, antioxidant supplementation has been proposed as a novel therapeutic strategy in this disease (Altindag et al. 2008). In this context, α-lipoic acid inhibited osteoclast differentiation (Kim et al. 2006), while lycopene was shown to reduce either oxidative stress or levels of bone turnover markers in postmenopausal women, reducing the risk of osteoporosis (Rao et al. 2007). These findings highlight the importance of nutrition in the maintenance of healthy bones, in particular with the benefit of D-vitamin supplementation. Noticeably, since the lowest incidence of osteoporosis in European countries has been reported in the Mediterranean area, it may be that the “Mediterranean lifestyle” that implies the consumption of foods containing a complex array of naturally occurring bioactive molecules with antioxidant, anti-inflammatory, and alkalinizing properties, can contribute to a “bone-sparing” effect (Puel et al. 2007).

### Oxidative stress, NADPH oxidase activity, and osteoclast function

Nox (NADPH oxidase) is a widely distributed enzyme in the body that catalyzes the generation of superoxide anion from molecular oxygen. This enzyme system can also generate O_2_^·–^ in osteoclasts (Yang et al. 1998; Steinbeck et al. 1994). One member of the Nox family, Nox4, is well expressed in kidneys, but is also abundant in the vascular system and in many other tissues and cells (Krause 2004), including osteoclasts (Yang et al. 2004), where its activity appears to be related to bone resorption. Abrogation of the normal level of Nox4 expression in osteoclasts inhibits both osteoclastic O_2_^·–^ production and bone resorption (Yang et al. 2001). An increase or decrease in superoxide anion production by osteoclasts is associated with, respectively, stimulated or inhibited osteoclastic bone resorption (Garrett et al. 1990; Berger et al. 2001; Ries et al. 1992).

Of note, O_2_^·–^ produced by osteoclasts in an extracellular compartment and even in the absence of other enzymes is capable of degrading bone matrix proteins such as osteocalcin, which break up into small peptides post exposure (Key et al. 1994).

### Oxidative stress, nitric oxide and bone remodeling

Nitric oxide (NO·) generated from arginine by NO synthase (NOS) is a free radical involved not only in vascular relaxation, platelet aggregation, neurotransmission, and immune system regulation (Moncada and Higgs 1993), but also in bone metabolism and function. Constitutive NOS (cNOS) is widely expressed in bone marrow stromal cells, osteoblasts, osteocytes, and osteoclasts (Helfrich et al. 1997), while inducible NOS (iNOS) has been observed in fetal bone (Hukkanen et al. 1999)—suggesting a role in skeletal development, but not constitutively in normal adult bone (Fox and Chow 1998). Osteoblasts stimulated with proinflammatory cytokines and/or endotoxin in vitro express iNOS (van't Hof and Ralston 1997), but it is still not clear whether osteoclasts express this enzyme. NO· can influence osteoblast or osteoclast activity and therefore bone remodeling.

Osteoblasts and osteocytes under mechanical strain and shear stress conditions produce large amounts of iNOS-mediated NO· that may augment bone gain by inhibiting prostaglandin-induced bone resorption (Ralston and Grabowski 1996). It has also been reported that osteocytes show a greater increase in NO· production as a result of mechanical loading than osteoblasts. This supports the theory that osteocytes are the main sensors and effectors of mechanical stress in bone (van't Hof and Ralston 2001).

Proinflammatory cytokines, such as IL-1 or TNF-α further increase NO· levels in bone tissues with subsequent potent inhibitory effects on osteoblast differentiation and growth. This may be eventually explained by the pro-apoptotic effects of NO· on osteoblasts (Armour et al. 2001), through a cGMP-dependent pathway (Mancini et al. 2000). This observation may explain the inhibitory effects of pro-inflammatory cytokines on bone formation.

Since chronic inflammation is a hallmark of ageing, this parameter may play a role in bone healing by altering bone fitness. This may explain the high incidence of hip fracture in elderly patients. Indeed, with the ageing of the population, hip fractures are a growing issue. At least half of hip fracture patients never regain their previous function (Stevens and Olson 2000), and post-hip fracture mortality at 1 year remains as high as 33% (Roche et al. 2005). The factors influencing recovery from hip fracture are poorly understood although persistent inflammation related to oxidative stress is a common feature in these patients.

When bone fracture occurs, the damaged tissues generate a remarkable amount of active radicals that are formed from the molecular interactions between embedded collagen strands and the mineral phase, and this phenomenon may facilitate bone healing (Symons 1996). As well, particular relationships between reactive oxygen species, hormones, and vitamins tie into bone metabolism. Estrogens, whose antioxidant activity is pivotal in protecting women of reproductive age from cardiovascular disease, stimulate osteoblastic activity through specific receptors, thus favoring bone growth. Therefore, primary estrogen deficiency in postmenopausal women can, through a loss of antioxidant potency, depress osteoblastic activity and indirectly enhance osteoclastic activity, leading to a reduction in skeletal mass and osteoporosis (Heaney et al. 1978; Raisz 1997). Another example, compensatory hyperparathyroidism, an unwanted consequence of chronic renal failure, can enhance osteoclastic activity and OCS generation, favoring bone resorption, which is characteristic of renal osteodystrophy (Freitag et al. 1978). The evidence suggests that OCS and antioxidants are involved in bone remodeling, although we cannot establish whether reactive species are the cause rather than the effect of the bone metabolic changes observed in many skeletal conditions and diseases.

More recently, many studies are supporting a role for the gut and its microbiome in the regulation of bone density and health. Direct modulation of the quantity of bacteria present (through use of antibiotics), as well as addition of bacterial substrates (prebiotics) and addition of beneficial bacteria (probiotics) can affect measures of bone health and calcium metabolism (reviewed in McCabe L et al. (McCabe et al. 2015)). However, there is still much to learn regarding our understanding of the signaling pathways that link the microbiome and gut to skeletal health. Future studies should be performed to pinpoint the mechanisms by which the microbiome regulates osteoblast and osteoclast activities.

In conclusion, oxidative stress is an important mediator of bone loss since increased free radical production overwhelms the natural antioxidants defense mechanisms, subjecting individuals to hyperoxidant stress and thus leading to osteoporosis or risk of hip fracture. Administration of antioxidants might protect bones from osteoporosis and help accelerating the healing of fractured bones.

## Role of oxidation in skin photoaging

Ageing is a multifactorial process characterized by metabolic, functional, and esthetic changes resulting from intrinsic genetically programmed processes and from environmental extrinsic factors (Helfrich et al. 2008; Thorin-Trescases et al. 2010). Photoaging is an accelerated form of ageing, specifically of the skin, resulting from exposure to ultraviolet (UV) light (Kligman 1989; Gilchrest 2013), causing the formation of deep wrinkles, loss of skin tone, dryness, elastic tissue clumping, pigmentary alteration, and exaggerated bruising (Yaar and Gilchrest 2012). Many of these clinical manifestations are a result of UV-induced changes in the dermis, the predominant target of UV radiation. Histopathological changes include alterations of elastin and the development of actinic elastosis, a hallmark of photoaging (Kligman 1969; Lewis et al. 2004). Actinic elastosis is characterized by an accumulation of elastotic material composed of abnormal elastin in addition to loss of fibrillar collagens and proteoglycans (Gilchrest 2013; El-Domyati et al. 2002; Sellheyer 2003; Weihermann et al. 2016). The key biological mechanism by which sun exposure leads to photoaging is the generation of reactive oxygen species (Gilchrest 2013; Yaar and Gilchrest 2012; Krutmann 2000)*.*

In the skin, reactive oxygen species can have deleterious effects on DNA, proteins, and lipids, particularly polyunsaturated fatty acids, leading to the formation of lipid oxidation products (LPO) which may contribute to dysfunction and skin damage (Vistoli et al. 2013). Ultraviolet A (UVA) is the largest source of reactive oxygen species, including the (non-radical oxidant) singlet oxygen (^1^O_2_) at the major contributor to photoaging. The skin has inherent defenses against UV-induced free radicals that include melanin, the pigment responsible for skin color, and constitutional repair processes present in damaged skin cells. Melanin is the first-line of defense against DNA damage at the surface of the skin. Pheomelanin, the melanin present in large quantities in red-haired individuals, is less protective than eumelanin, the melanin seen in blonde, brown, and black-haired individuals. However, melanin alone cannot entirely prevent skin damage even in those with very dark skin color. A second category of defense is repair processes which remove damaged biomolecules before they can accumulate and alter cell metabolism.

Oxidative stress and subsequent tissue damage occurs when these lines of defense are overwhelmed. This point of saturation for the cell’s defense mechanisms may differ depending on the individual skin type and vulnerability (Battie et al. 2014; Rinnerthaler et al. 2015). Regardless, topical and oral antioxidants may combat photoaging by disrupting the molecular processes that promote reactive oxygen species generation. These antioxidant supplements are not FDA-regulated and consistency in recommendation is lacking.

### Structure of the skin

The skin is comprised of three main layers including the epidermis, dermis, and subcutaneous fat. The epidermis is the most superficial layer and is composed of stratified squamous cells or keratinocytes in addition to melanocytes and Langerhans cells, the immune cells of the epidermis. The dermis can be found just beneath the epidermis. This is the layer of the skin predominately targeted by the process of photoaging. The dermis is a flexible connective tissue network primarily comprised of collagen and elastin fibers embedded in a ground substance matrix that supports nerves, sensory receptors, lymphatic channels, and vasculature (Robinson 2015). Collagen comprises the majority of the dermis with collagen type I constituting 75% of this total. The predominant role of collagen is to impart strength and structure to the skin. Elastic fibers are synthesized by fibroblasts and keratinocytes and comprise approximately 3% of the dermis. The primary role of elastic fibers is to confer the skin with elasticity and resilience. The third component of the dermis is the ground substance. This material fills the spaces between collagen and elastin fibers and adds resilience to the skin. It is comprised of glycosaminoglycans (hyaluronic acid and dermatan sulfate) and glycoproteins (Robinson 2015).

### UV-induced production of reactive oxygen species

Sunlight, the source of UV radiation, is divided into three main types based on wavelength. UVC (200–280 nm) is filtered by the ozone layer, UVB (280–320 nm) is mainly absorbed by the epidermis, and UVA (320–400 nm) deeply penetrates into the dermis. Aside from the direct reaction of UVB photons with DNA in the epidermis, an important consequence of skin irradiation by UVR (mainly UVA which represents more than 95% of solar radiation) is the generation of reactive oxygen species. This relationship between UV exposure and reactive oxygen species generation has been demonstrated by in vivo studies using markers of antioxidant activity such as ferritin. UVA exposure increases ferritin expression in basal and suprabasal keratinocytes in the epidermis and interstitial cells in the dermis suggesting an increase in oxidative stress induced by UVA exposure. Reactive oxygen species generation results from interactions between UV photons and endogenous photosensitizers (porphyrins, riboflavin, quinines, trans-urocanicacid) that become excited and react with O_2_ to produce reactive oxygen species such as superoxide anion radical(O_2_^·–^) and hydroxyl radical (·OH) non-radicals such as singlet oxygen (^1^O_2_) and hydrogen peroxide (H_2_O_2_) (Wenk et al. 2001; Panich et al. 2016). The singlet oxygen anion induces guanine moiety oxidation followed by structural rearrangement and 8-oxo-7,8-dihydroguanine (8-oxo-G) and 8-oxo-7,8-dihydro-2′-deoxyguanosine (8-oxo-dG) formation, which are biochemical markers of UVA-induced DNA damage (Panich et al. 2016). Protective pathways include both antioxidative and base excision repair (BER) pathways, but these pathways may become overwhelmed. In such instances, UVR-induced reactive oxygen species can lead to photoaging by varied mechanisms (Panich et al. 2016).

### Skin photoaging pathophysiology

The skin is largely exposed to ambient UV-irradiation placing it at high risk for photooxidative damage. Oxidative stress leads to clinical signs of photoaging via a variety of different molecular mechanisms that converge on altered epidermal and dermal DNA, lipids, and proteins (Table 1).

**Table 1:** Contrast in histologic appearance of intrinsic ageing vs. photoaging (El-Domyati et al. 2002; Bosset et al. 2003)

	Intrinsic ageing	Photoaging
Epidermal basal layer	Single layer of cuboidal cells with 1 melanocyte per 5–10 basal keratinocytes intermixed with Merkel cells	
	No apoptosis	Apoptosis of epidermal basal keratinocytes
Cellular infiltrate	One layer of CD1a + cells found in lower epidermis	
	Decreased Langerhans cell numbers	
	Memory CD4+ T cells around superficial dermal blood vessels	CD1a+ cells distributed in two superposed layers in lower and mid-epidermis. CD1a+ cells more irregular and less dendritic
	Increased number of CD1a+ and protein S100 (PS100)+ dendritic cells	
	Increased mast cells and macrophages, especially in areas of elastosis	
	Decreased Langerhans cell numbers	
	Increased CD4+ T cells after acute drop in days following sun exposure	
Epidermal thickness	Minimal to no thinning: Approximately 0.1 mm (50–100 cell layers thick)	Atrophy/thickening
Dermal collagen	Decreased: diminished synthesis and/or increased degeneration (via abnormally increased expression of MMPs in aged skin)	Decreased: diminished synthesis and increased degeneration (via UV-induced up-regulation of MMPs)
Fibroblasts	Intact	Apoptotic in the upper dermis
Elastic fibers	Dark purple staining of elastic fibers. This correlates with loss of elastic tissue primarily in fine subepidermal elaunin network. In the reticular dermis, the elastic network may be irregularly thickened, fragmented, and disorganized	Solar elastosis: loss of eosin staining with resulting bluish color in upper dermis. This correlates with increased deposition of thickened, tangled, and granular amorphous elastic structures

Clinical features of photoaging include deep wrinkling, dyspigmentation, sallowness, telangiectasia, laxity, and a leathery appearance (Helfrich et al. 2008). These clinical changes are the result of UV-induced microscopic changes occurring across skin levels, but predominantly at the level of the dermis. There are also UV-induced mutations in keratinocytes and melanocytes and UV-induced apoptosis of the stem cells in the basal layer of the epidermis in addition to the hair bulge, which contributes to the clinical features of slow wound healing and dyspigmentation (Gilchrest 2013). Epidermal thickening or atrophy may also occur (Beckman and Ames 1998).

In the dermis, there are a number of UV-induced changes that eventuate in, among others, skin wrinkling and loss of skin turgor and elasticity. Collagen degradation is accelerated with exposure to UV light. The decline in collagen with age is due at least in part to the increased activity of matrix metalloproteinases (MMPs), collagenase, 92 kd gelatinase, heme oxygenase-1, superoxide dismutase-2, and stromelysin (Battie et al. 2014). The activity of MMPs increases with exposure to UV light, increasing the destruction of existing collagen. There is also decreased production of de novo collagen. In vitro models report the disappearance of dermal fibroblasts in the upper part of the dermal equivalent within 48 h following UV exposure via an apoptotic process (Battie et al. 2014). The progressive loss of fibrillar collagen and collagen VII from the dermis leads to a large decrease in volume and strength with resultant wrinkling, easy bruising, and torpid wound healing (Gilchrest 2013).

Elastosis, or the accumulation of partially degraded elastin fibers in the dermis, is another hallmark of photoaging (Gilchrest 2013). This is the result of UV-induction of destructive proteins such as lysosomal protease cathepsin K, the most potent of the elastin-degrading enzymes, increases in deposits of lysozyme, and alternative splicing (Gilchrest 2013). Lysozyme deposits on elastic fibers are seen in sun-damaged regions with the number of lysozyme-containing elastin fibers appearing to correlate with the degree of sun damage. It has been reported that lysozyme exerts its elastin-damaging effects by binding to fibers and inhibiting the proteolytic degradation of altered fibers by elastases (Battie et al. 2014). Thus, the occurrence of elastosis results from a reduction in functional elastin and an increase in non-functional elastin. In addition, UV radiation may induce altered splicing and expression of tropoelastin isoforms that contain exon 26A, which code for a highly lipophilic segment. These isoforms may alter the structural and biological properties of elastin and affect normal elastic fiber formation (Weihermann et al. 2016).

Additional manifestations of UV-induced damage include detrimental DNA deletions. On average, skin cells exhibit some 4977 base pair deletions. In photoaged individuals, this deletion is up to 10-fold more prevalent. This compromises mitochondrial integrity and energy production, and increases mitochondrial superoxide production, which is believed to be related to the clinical signs of photoaging (Gilchrest 2013).

The full extent of oxidative stress responses as experienced by different skin types remains unexplored, but clinical manifestations suggest that mechanisms for damage and repair may differ. Photoaging in more fair-skinned patients manifests with deep wrinkles and in darker-skinned patients manifests as dyspigmentation. This may result from racial variation in individual and aggregated melanosome distribution. For example, in Asians, there is a mix of individual (about 60%) and aggregated (about 40%) melanosomes that differs from European skin in which aggregated melanosomes are the majority at 85% (Battie et al. 2014). There appears to be a difference in the quantity of UV-induced DNA mutations in different skin tones with in vitro work demonstrating an inverse relationship between skin color and UV-induced skin response. There is a dose-dependent accumulation of DNA damage, throughout the entire epidermal layer and upper dermis of light, intermediate, and tan skin tones. In brown and dark skin tones, however, DNA damage is detected exclusively in suprabasal epidermal layers.

Although variations in oxidative stress by skin type remain largely unexplored, the general mechanisms by which UV-induced oxidative stress leads to clinical signs of photoaging are more established. Oxidative stress incurred by human skin fibroblasts after UVR enhances expression of cell cycle inhibitors p16INK4A and p53/p21WAF1/CIP1, which eventually result in premature senescence of fibroblasts. These fibroblasts are not only key for collagen production, but also function as a key component of the skin microenvironment. This fibroblast dysfunction destabilizes the microenvironment vital for skin stem cells (Panich et al. 2016). UV-induced overexpression of c-Myc is correlated with depletion of epidermal stem cells, causing them to over proliferate, move out of their niche, and differentiate (Panich et al. 2016).

Reactive oxygen species also activate signaling pathways in resident fibroblasts such as growth factor receptors, MAPK, or redox-sensitive transcription factors, leading to secretion of metalloproteases that degrade the proteins of extracellular matrix (ECM), including collagen. Reactive oxygen species indirectly provoke DNA oxidation and mutations, particularly the formation of 8 oxo-deoxyguanosine (8-oxo-dG) in guanine bases, which may be involved in skin cancer development (Wenk et al. 2001; Wang 2011). Reactive oxygen species may also activate the p53-p21 axis, Akt-MAPK, ATM pathways in addition to the forkhead homeobox type O (FOXO) transcription factors in various types of stem cells by influencing telomere length, thus accelerating the ageing process in skin cells (Panich et al. 2016). Furthermore, UV-radiation results in activation of cell surface receptors leading to the activation of MAP-kinase p38, c-Jun amino-terminal kinase (JNK) and extracellular signal-regulated kinase (ERK) and the recruitment of c-Fos and c-Jun. This leads to the expression of MMP 3 and 9 in fibroblasts and keratinocytes (Rinnerthaler et al. 2015). Activator protein (AP)-1 inhibits transforming growth factor (TGF)-β responsible for collagen production and up-regulates the level of MMPs responsible for collagen degradation. AP-1 levels become elevated and remain elevated for at least 24 h following UV radiation. During this time frame, MMPs are up-regulated and collagen and ECM damage ensues (Helfrich et al. 2008).

In addition to DNA, lipids are a predominant target of the reactive oxygen species oxidative attack, particularly polyunsaturated fatty acids (PUFA) that are present in the plasma membranes of fibroblasts. PUFA oxidation generates lipid oxidation products (LPO), including aldehydes such as 4-hydroxynonenal (HNE), malondialdehyde (MDA), acrolein, all of which form adducts on proteins, leading to progressive dysfunction and accumulation as modified material (carbonyl stress) (Vistoli et al. 2013). The role of carbonyl stress evoked by LPO in skin ageing is not yet clarified. However, aldehyde adducts (HNE-, acrolein-adducts) are detected in the lower epidermis and in the dermis, and could contribute to fibroblast dysfunction and ECM modification (Larroque-Cardoso et al. 2015).

### Preventing or minimizing photoaging

Unlike the process of intrinsic ageing that cannot be halted, steps may be taken to reduce the damage from non-chronological factors. The cell has its own endogenous pathways to protect against oxidative damage. This includes enzymatic antioxidants such as superoxide dismutase (SOD), catalase (CAT), glutathione peroxidase (GPx), glutathione reductase, and thioredoxin reductase (Panich et al. 2016). Nuclear factor erythroid-2 related factor 2 (Nrf2) has also been implicated as a key regulator of redox homeostatic genes involved in skin adaptation to oxidative stress, with its depletion compromising the ability of a given cell to protect against photo-oxidative damage (Panich et al. 2016). When these pathways are functional and faced with low-level oxidative stress, the redox balance is maintained. However, extrinsic exposures may overwhelm endogenous protective mechanisms leading to oxidative tissue damage and thus photoaging.

Supplemental topical and dietary antioxidants may offset the damage caused by an overwhelmed antioxidative system. Common examples of antioxidants include vitamin C (ascorbic acid), vitamin E (alpha-tocopherol), beta-carotenes, and a variety of phytochemicals, particularly polyphenols derived from plant-based foods and herbal products. Given their mechanism of action, however, effective antioxidants would need to have a very high antioxidative capacity and reactivity, to be present in extremely high concentrations, to be stable in their final formulation, and (if topical) to be able to penetrate the skin barrier (Wang et al. 2011).

Vitamin C is a common, water-soluble, antioxidant found in the diet. It functions as a cofactor for many enzymatic reactions including the crosslinking of collagen. It also scavenges free radicals via donation of its electron and formation of a comparatively inert radical. It can then either be further reduced or can react further to become dehydroascorbic acid. Vitamin C may also be involved in a redox cycle that can re-reduce membrane-bound Vitamin E after it has been oxidized in acting as a chain-breaking antioxidant. Vitamin E is found in plant oils and has the ability to stop ongoing lipid peroxidation chain reactions via reduction of lipid peroxl radicals to hydroperoxides. In this process, it is itself transformed into a comparatively inert radical that may be further detoxified by ascorbate or glutathione. Topical application of vitamin E further works by decreasing MMP-1 transcription levels and pyrimidine dimer photoproduct formation (Rinnerthaler et al. 2015). Beta-carotene, a precursor to vitamin A, also works by scavenging radicals and inhibiting lipooxygenases capable of producing reactive oxygen species. The peroxyl radical is directly added to its backbone forming an epoxide that is later decomposed (Rinnerthaler et al. 2015).

Protection from oxidatively induced damage is primarily via application of a broad-spectrum sunscreen with UVA and UVB protection. Particular emphasis for photoaging is placed on UVA (320 to 400 nm) protection given its central role in cutaneous reactive oxygen species production (Wang et al. 2011). UVB (290 to 320 nm) is characterized by superficial penetration and is primarily responsible for sunburns and direct DNA damage. While antioxidants do exist as standalone ingredients in oral and topical formulations, manufacturers have begun to include antioxidants in sunscreens with the claim of added oxidative damage protection (Wang et al. 2011). In theory, this provides two layers of protection: a “passive” layer and an “active” layer. The “passive” layer is comprised of UV filters that absorb or reflect UV rays and the “active” layer is comprised of antioxidants that neutralize any reactive oxygen species generated despite the passive layer. Whether such a system works upon application to the skin is unknown.

Quantification of antioxidant effectiveness has many limitations and varied assays exist for such purpose. The technical application, uses, and limits of such assays have been described elsewhere (Pinti et al. 2016). There is no validated rating system or single assay used between and among products labeled as antioxidants and, thus, the ability of a consumer to draw conclusions about effectiveness is limited. A common assay is the radical skin protection factor (RSF). This is an ex vivo technique using porcine skin that directly measures protection against UVA-induced free radical formation. It is calculated from the ratio of free radicals in unprotected skin to those in protected skin with the radicals quantified using electron spin resonance (ESR) spectroscopy (Wang et al. 2011). The proportion of protection provided by antioxidants can be determined by the antioxidant power (AP) which compares oxidation protection provided by antioxidants versus UV filters. The AP value is generated in vitro utilizing an equation comprised of the constant reduction amplitude, the quantity of reduced free radicals, the characteristic weight of the antioxidant product, and the reduction time. The resulting value is expressed in antioxidative units (AU) with ascorbic acid utilized as the standard (Wang et al. 2011).

Wang et al. used AP to determine the benefit of supplementing sunscreens with antioxidants and found low to zero AP units in many of the evaluated sunscreens indicating little additional protection from antioxidant additives. Of the 12 products tested, five products had an RSF over 15 (range 16.5–27). Of these five, the most common UV filters were octocrylene, titanium dioxide, and butyl methoxydibenzoylmethane. Ten of the 12 products had an antioxidant power of 0. Those with any measureable antioxidant power had multiple antioxidant additives and perhaps multiple combinations may offer better antioxidant protection. (Wang et al. 2011).

Clinical studies have demonstrated an ability of sunscreens to minimize some features of photoaging. A study of 35 adults demonstrated solar elastosis was significantly decreased (*p* = 0.0001) by at least twice-daily application of a sunscreen consisting of 7% octylethoxycinnamate, 6% oxybenzone, and 5% octyl salicylate over 2 years (Boyd et al. 1995). Another study reported a highly photostable sunscreen (high UVA protection factor 22) was able to protect against multiple cellular markers of photoaging, sunburn cells, Langerhans cell depletion, cyclobutane pyrimidine dimer formation, and p53 expression. Clinical signs of photoaging from baseline to the end of the trial were reduced by 24% in the daily sunscreen group compared to the discretionary sunscreen group (relative odds 0.76, 95% confidence interval (0.59–0.98) (Young et al. 2016).

Stand-alone antioxidants have been studied within various animal and human tissue models demonstrating benefit in certain markers of reactive oxygen species damage. However, the real life efficacy of these products may be dramatically different from those noted in these studies largely due to study designs which do not replicate real world conditions in regards to time and location of application.

Photoaging is largely due to ultraviolet-induced reactive oxygen species and is distinct from intrinsic ageing. Intrinsic ageing is characterized by skin thinning, laxity, and fine wrinkling whereas photoaging is characterized by dryness, dyschromia, telangiectasia, and deep wrinkling. (Rui Yin and Hamblin 2015). UVA-radiation is primarily responsible for photoaging due to its deep penetration into the skin with effects on collagen, elastin, and the extracellular matrix. Topical and oral antioxidants have been utilized with the goal of quenching reactive oxygen species, but whether such products prevent photoaging in a clinically meaningful way remains unknown. Given the large role of UVA in photoaging, however, it is generally recommended that efforts at prevention of photoaging should incorporate use of a broad-spectrum sunscreen.

## Cataracts

The cause of cataracts, which is when the lens of the eye becomes clouded and impairs eyesight, stems from many different sources, yet many of these sources involve the unbalanced production of free radicals in common. In the eye lens, free radicals target the polyunsaturated fatty acids and lens crystallin proteins leading to the initiation of cataracts (Thiagarajan and Manikandan 2013). Studies examining whether dietary antioxidants offer protective effects against cataracts are promising, but generally show mixed results.

Vitamin C (ascorbic acid) has strong antioxidant properties and has been shown to protect against DNA damage by quelling oxidative stress. Yet, in the presence of transition metals, vitamin C, paradoxically, contributes to DNA damage (Cai et al. 2001). Dietary supplementation of vitamin C has been associated with reduced risk of developing age-related cataracts (Yoshida et al. 2007; Jacques and Chylack 1991; Hankinson et al. 1992). Though its effectiveness is still up for debate, as other long-term studies showed that dietary supplementation of vitamin C had no effect on cataract development (Christen et al. 2010). Moreover, vitamin C has been shown to effectively protect against certain types of cataracts such as nuclear cataracts, but not others such as cortical or posterior subcapsular cataracts (Tan et al. 2008).

Vitamin E is another dietary antioxidant, however clinical trials have shown that dietary supplementation of vitamin E did not prevent development or slow progression of age-related cataracts (McNeil et al. 2004) or did vitamin A (Chasan-Taber et al. 1999). Additionally, diet-derived carotenoids from fruits and vegetables showed a modest effect in decreasing the risk of cataracts over the course of an 8-year study (Brown et al. 1999) and 12-year study (Chasan-Taber et al. 1999). However, not all carotenoids confer protection against cataracts, only lutein and zeaxanthin had a measurable effect in both studies.

## Age-related macular degeneration

### The APOE/cholesterol paradox of age-related macular degeneration

Age-related macular degeneration (AMD) is a common, complex disease that results from interplay of genetic and environmental risk factors. Polymorphisms in the complement factor H (CFH) and in Apolipoprotein E (APOE) account for a large part of the genetic risks to AMD (Swaroop et al. 2007). Age, smoking, and obesity are the main environmental risk factors (Chakravarthy et al. 2010). The presence of large drusen, sizeable extracellular deposits of lipids and glycoproteins (Farkas et al. 1971) in Bruch’s membrane (BM), are a major risk factor for debilitating late AMD (Klein et al. 2004). Large drusen in the absence of vision loss is therefore considered the defining feature of intermediate AMD. Choroidal neovascularisation (wet AMD), or an extending lesion of the retinal pigment epithelium (RPE) and photoreceptors (geographic atrophy, GA) characterize late AMD (Sarks 1976). Intermediate AMD affects more than 150 million people worldwide and 10 million patients suffer from late AMD (Wong et al. 2014).

Yet, our understanding about complex interactions of risk factors and AMD formation is relatively unknown. Early and late forms of AMD show chronic, non-resolving subretinal accumulation of mononuclear phagocytes (MP) (Levy et al. 2015a; Combadiere et al. 2007; Sennlaub et al. 2013; Lad et al. 2015; Gupta et al. 2003). MPs include monocytes (Mo), resident macrophages (rMφ) such as microglial cells (MC), and monocyte-derived inflammatory macrophages (iMφ) that arise during inflammation (Nathan and Ding 2010). While acute MP infiltration likely helps resolve large drusen (Klein et al. 2002; Smith et al. 2010), their chronic infiltration contributes significantly to the pathogenesis, as it can cause considerable collateral damage and fuel further inflammation (Nathan and Ding 2010). MPs plays a critical role in pathological choroidal neovascularisation (CNV) (Tsutsumi et al. 2003; Sakurai et al. 2003) and photoreceptor degeneration (Combadiere et al. 2007; Sennlaub et al. 2013; Rutar et al. 2012; Suzuki et al. 2012; Kohno et al. 2013; Cruz-Guilloty et al. 2013; Hu et al. 2015; Eandi et al. 2016) in advanced AMD (Moore and Tabas 2011).

Large drusen and atherosclerotic lesions contain similar extracellular proteins (e.g., vitronectin, complement factors, and apolipoprotein E) and important amounts of cholesterol and cholesterol esters (Moore and Tabas 2011; Pikuleva and Curcio 2014; Hageman and Mullins 1999; Mullins et al. 2000; Curcio et al. 2001). These observations and experimental data led to the hypothesis that drusen lesions develop secondary to a deficit in the reverse cholesterol transport (RCT) similar to atherosclerotic plaques (Pikuleva and Curcio 2014; Malek et al. 2005; Ong et al. 2001). The RCT pathway directs excess cholesterol by high-density lipoproteins (HDL) to the liver for elimination, to avoid toxic overload of cholesterol in peripheral cells. Similarly, in atherosclerosis it is well established that low levels of HDL and high levels of non-HDL cholesterol participate in cholesterol accumulation and plaque formation (Moore and Tabas 2011). Accordingly, APOE^−/−^ mice have extremely high lipid levels and massive amounts of cholesterol-rich β-VLDL and spontaneously develop atherosclerotic lesions (Meir and Leitersdorf 2004). Interestingly, macrophage APOE plays an important role in preventing the plaque formation (Van Eck et al. 1997; Fazio et al. 1997). APOE^−/−^ mice also accumulate lipid debris in BM proposed to be similar to drusen (Ong et al. 2001), supporting the hypothesis that deficient RCT is also involved in drusen genesis.

### The AMD paradox

Taken the similarities of large drusen and atherosclerotic plaques, one might expect a similar deregulation of the RCT with both diseases. Curiously, the reverse is the case: high levels of HDL, protective against atherosclerosis, are associated with AMD (Klein et al. 1993; Klein et al. 1997; Klein et al. 2003; Delcourt et al. 2001; van Leeuwen et al. 2004; Paun et al. 2015). A polymorphism of the ABCA1, linked with low HDL and impaired RCT, has been shown to be protective against advanced AMD (Chen et al. 2010). APOA-I levels are elevated (not decreased) in the vitreous (Koss et al. 2014) and serum (Paun et al. 2015) of AMD patients. Most importantly, the APOE*4*-isoform, associated with decreased APOE levels (Riddell et al. 2008; Bales et al. 2009; Sullivan et al. 2011; Levy et al. 2015b) (see below) and impaired reverse cholesterol transport (Heeren et al. 2004; Mahley et al. 2009), protects against AMD (McKay et al. 2011). In reference to the “French paradox” (that summarizes the apparently paradoxical epidemiological observation that the frequency of ischemic heart diseases in France is relatively moderate despite having a diet rich in cholesterol (Richard 1987)), one might therefore speak of an “AMD-paradox” describing the seemingly paradoxical association of markers of enhanced RCT with AMD, despite the obvious morphological and molecular similarities between cholesterol-rich drusen and atherosclerotic lesions.

### AMD and apolipoprotein E

APOE is expressed in the liver and is the main lipoprotein of the brain and the retina (Mahley and Rall 2000; Anderson et al. 2001). It is strongly secreted by hepatocytes, but also in the RPE (Ishida et al. 2004) and in mononuclear phagocytes (MPs), such as Mφs and MCs (Levy et al. 2015a; Peri and Nusslein-Volhard 2008). As mentioned above, APOE plays a major role in macrophage lipid efflux and RCT in conjunction with APOA-I (Mahley et al. 2009; Mahley and Rall 2000; Peri and Nusslein-Volhard 2008). APOE, APOA-I, and cholesterol can also influence innate immunity. Cholesterol crystals can activate the innate immunity receptor cluster, formed by toll-like receptors (e.g., TLR2, TLR4) and obligate co-receptors (such as CD14) that activate Myd88, NFκB and induce inflammatory cytokines (CCL2, IL-6, TNFα, etc.). APOE and APOA-I can also bind and neutralize lipophilic TLR ligands (such as β-amyloid and LPS) and inhibit the induction of inflammatory cytokines (Azzam and Fessler 2012). But an excess of APOE and APOA-I can also activate the TLR2-TLR4-CD14-dependent IIRC, in the absence of extracellular ligands: Under physiological conditions, the different components of the IIRC of Mφs are separated from each other, as some elements cluster to cholesterol-rich membrane domains called lipid rafts (e.g., CD14) while others are located in the non-raft plasma membrane (e.g., TLR2 and TLR4) (Pfeiffer et al. 2001). IIRC ligands, such as bacterial lipopolysaccharides, overcome this separation, as they bind to the extracellular domains of both co-receptors (CD14 and TLRs), bringing the receptors and their intracellular domains closely together, which activates Myd88, NFκB and induces the transcription of inflammatory cytokines (Schmitz and Orso 2002). However, in the absence of IIRC ligands, excessive APOA-I and/or APOE can extract enough cholesterol from the lipid raft to lift the physiological separation of the IIRC co-receptors and trigger the intracellular signaling (Levy et al. 2015a; Smoak et al. 2010). In sterile inflammation, excessive APOA-I and APOE can thereby induce inflammatory cytokines such as IL-6 and CCL2 (Levy et al. 2015a; Smoak et al. 2010).

In AMD, APOA-I levels are elevated in the vitreous (Koss et al. 2014) and serum (Paun et al. 2015) and APOE accumulates in drusen (Klaver et al. 1998) and is strongly expressed by subretinal MPs (Levy et al. 2015a). In mice, we showed that subretinal MPs of *Cx3cr1* ^*GFP/GFP*^-mice that develop subretinal inflammation and cardinal features of AMD (Combadiere et al. 2007) express similar high levels of APOE (Levy et al. 2015a), but also IL-6 (Levy et al. 2015a) and CCL2 (Sennlaub et al. 2013). Increased levels of CCL2 and IL6 are also observed in late AMD (Sennlaub et al. 2013; Jonas et al. 2010; Seddon et al. 2005; Chalam et al. 2014). *ApoE* deletion in *Cx3cr1* ^*GFP/GFP*^-mice nearly completely prevented age- and stress-induced pathogenic subretinal MP accumulation (Levy et al. 2015a). Mechanistically, APOE-induced IL-6 release from MPs represses RPE immune-suppression and prolongs subretinal MP survival and accumulation (Levy et al. 2015a). Additionally, APOE-dependent increase of CCL2 recruits pathogenic inflammatory CCR2^+^ monocytes to the subretinal space (Sennlaub et al. 2013). Excessive APOE, and possibly APOA-I, can thereby induce chronic, pathogenic MP accumulation due to decreased MP elimination (IL-6) and increased MP recruitment (CCL2).

In humans, the *APOE* gene has three common genetic variants (*APOE2*, *APOE3*, and *APOE4*) that lead to six possible diplotypes. *APOE4*, believed to be the ancestral form (Raichlen and Alexander 2014), evolved to *APOE3*, and *APOE3* to *APOE2* due to two polymorphisms (rs429358 and rs7412, respectively) that are imbedded in a well-defined CpG island, and lead to two cysteine-arginine interchanges at residues 112 and 158 (Yu et al. 2013). In most populations, *APOE3* is now the most common isoform (80% frequency) compared to *APOE4*- (14% frequency) and *APOE2*-isoforms (6% frequency). The *APOE* isoforms are associated with several common, age-related diseases. Notably, the *APOE4*-allele is the most important genetic risk factor for Alzheimer’s disease and a risk factor of atherosclerosis, while *APOE2* is protective for Alzheimer disease and atherosclerosis (except if associated with type III familial hyperlipiproteinemia) (Mahley et al. 2009; Herz and Beffert 2000; Davignon et al. 1988). Curiously, the association with AMD is inversed: *APOE2*-allele carriers are at increased risk for developing late AMD (OR = 1.83 for homozygote carriers), while the *APOE4*-allele protects for AMD (OR = 0.72 per haplotype) compared to the most common *APOE3*-allele recently confirmed in a worldwide study comprising 20,000 subjects (McKay et al. 2011). This association was found for both clinical forms of late AMD, but not for early/intermediate AMD (Paun et al. 2015), suggesting the APOE isoforms are not primarily implicated in the lipid accumulation in drusen genesis. Indeed, the targeted replacement mice expressing the human APOE4 (TRE4), and not APOE2 (TRE2), accumulate most lipids in Bruchs membrane compared to APOE3-expressing TRE3 mice (Malek et al. 2005), although *APOE4-*allele plays a protective role in human AMD (McKay et al. 2011). Similar, lipid deposits are also observed in APOE^−/−^ mice (Ong et al. 2001), but AMD is associated with increased APOE immunoreactivity (Levy et al. 2015a; Anderson et al. 2001; Klaver et al. 1998) in the human disease. These results suggest that the reasons for the association of the APOE isoforms with AMD are not due to a decreased capacity to evacuate cholesterol from Bruchs membrane.

The *APOE* isoforms are also associated with differences in APOE abundance: APOE plasma concentrations are significantly higher in homozygous *APOE2*-diplotype carriers and lower in *APOE4*-carriers compared to the *APOE3* homozygous diplotype (*E2/E2*: 13.8 mg/dl; *E3/E2*: 7.3 mg/dl; *E4/E2*: 6.7; *E3/E3*: 5.5 mg/dl; *E3/E4*: 5 mg/dl; *E4/E4*: 4.4 mg/dl) (Smit et al. 1988). The increased APOE concentrations associated with the *APOE2* isoform are likely due to its decreased affinity for the LDL receptor and impaired clearance (Mahley and Rall 2000), but also to increased transcription in certain cell types (astrocytes and Mφs), due to the loss of a CpG site present in the *APOE3*-alleles (Levy et al. 2015b; Yu et al. 2013). *APOE2*-carriers also display higher APOE concentrations in cerebrospinal fluid, brain tissue, and the retina (Riddell et al. 2008; Bales et al. 2009; Levy et al. 2015b). Compared to the *APOE3*-allele, the *APOE4*-allele is transcribed similarly in neurons, astrocytes, and Mφs (Levy et al. 2015b; Yu et al. 2013), but its protein concentration in plasma (see above), CSF, brain, and retina are decreased (Riddell et al. 2008; Bales et al. 2009; Sullivan et al. 2011; Levy et al. 2015b). The structural changes in the APOE4 protein also lead to diminished association with HDL (Dong and Weisgraber 1996) and impaired reverse cholesterol transport (Heeren et al. 2004; Mahley et al. 2009).

Using the targeted replacement mice expressing the human APOE isoforms (TRE2, TRE3, and TRE4), we recently showed that the increased levels of APOE in MPs of *TRE2*-mice, activates the IIRC and induces IL-6, and CCL2 (Levy et al. 2015b) similar to *Cx3cr1* ^*GFP/GFP*^-mice (Levy et al. 2015a). As a consequence, *TRE2*-mice develop subretinal MP accumulation, photoreceptor degeneration, and exaggerated choroidal neovascularization akin to AMD (Levy et al. 2015b). In the context of APOE-dependent subretinal inflammation in *Cx3cr1* ^*GFP/GFP*^-mice, the *APOE4*-allele led to diminished APOE tissue levels and CCL2 levels and protected *Cx3cr1* ^*GFP/GFP*^-mice against harmful subretinal MP accumulation observed in *Cx3cr1* ^*GFP/GFP*^ *TRE3* mice (Levy et al. 2015b).

A possible pathogenic role of excessive cholesterol extraction and IIRC activation in AMD is also supported by the observation of increased APOA-I levels in AMD patients (Paun et al. 2015; Koss et al. 2014), the protective effect of an ABCA1 polymorphism (associated with possibly impaired RCT) (Chen et al. 2010), and clinical studies showing that statins (that inhibit cholesterol synthesis) can accelerate the progression to late AMD (VanderBeek et al. 2013).

Our study also sheds an interesting light on the puzzling differences of the APOE isoform association with AMD (McKay et al. 2011) and Alzheimer disease (Mahley and Rall 2000), two major age-related neurodegenerative diseases: In Alzheimer disease, the *APOE4*-allele is associated with greater β-amyloid burden, possibly due to decreased APOE tissue concentrations and reduced efficacy in clearance of β-amyloid clearance via multiple pathways (Bales et al. 2009; Mahley et al. 2009). Accordingly, Cx3cr1^−/−^mice that express increased amounts of APOE in MPs, including MCs (Levy et al. 2015a), are protected against beta-amyloid deposition in Alzheimer disease mouse models (Lee et al. 2010b).

While a lack of APOE and RCT might play a pathogenic role due to reduced efficacy of cholesterol and β-amyloid clearance in atherosclerosis (Mahley et al. 2009) and Alzheimer disease (Bales et al. 2009; Mahley et al. 2009), excessive APOE and RCT might be responsible for IIRC activation and chronic inflammation in AMD. These fascinating differences might help explain the APOE/cholesterol paradox of age-related macular degeneration.

## Conclusions

**Fig. 5:**
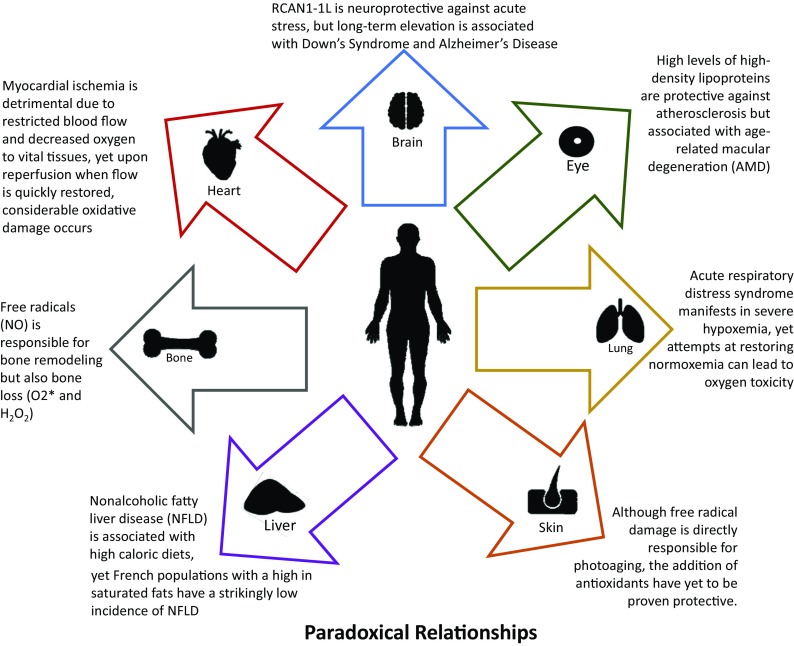
Paradoxical relationships in the oxygen paradox and age-related, organ-based diseases. The figure points out just some of the paradoxical relationships between substances (free radicals, antioxidants) or gene products (RCAN1, HDL), oxygen itself, sunlight, fatty foods, hypoxia and reperfusion, etc. at low versus high concentrations, doses, or exposures that have been discussed in this review, and common age-related diseases of major organs. Please note that space limitations preclude a truly exhaustive listing or pictography

This review explores the phenomenon of paradoxical relationships in biology, particularly as they relate to ageing and the etiology and/or progression of age-related diseases (Fig. 5).

Our particular focus is on the oxygen paradox as a means of exploring the potential underlying mechanisms responsible for the French paradox. We discuss adaptive homeostasis and how environmental signals, such as sub-toxic, non-damaging levels of oxidants can trigger the body’s defense mechanisms to confer protection against further assaults. We also examine how the decline of adaptive homeostasis associated with age brings with it a decreased ability to respond to stress. This review also discusses age-associated dysfunction in major human organs where we explore several mechanisms of defense and degeneration. The common theme in age-related oxidative damage is an imbalance in the redox state, accompanied with increased oxidative stress. For example, in bones oxidative stress is a major mediator of bone loss, as well as in the skin where reactive oxygen species damage is responsible for photoaging. Similarly, neurodegenerative diseases and ocular degeneration also have their roots in oxidatively generated damage.

The oxygen paradox, specifically the dangers of imbalanced oxygen or oxygen radical levels is clearly illustrated in the lungs and heart. In the lungs, the oxygen paradox was clearly experienced during the early development of treatments for ARDS-related hypoxemia with high FIO2 volume ventilation used to try to regain normoxemia. It was quickly found, however, that a large influx of oxygen would often result in oxygen toxicity and lead to worsening of the lung injury. A balance was needed to find a “safe” and low FIO_2_ level that would result in an effective treatment of ARDS. In the heart, the oxygen paradox is illustrated by the pathology of myocardial ischemia where blood flow is initially restricted, but then is quickly restored leading to reperfusion injury. During and after reperfusion is a time of considerable oxidative stress that leads to extensive cellular damage. However, should a myocardial infarction actually occur, treatment is most successful if administered in the small-time window pre-reperfusion. The detrimental reduction in available oxygen as a result of myocardial ischemia followed by the toxicity of high oxygen exposure after reperfusion is a prime (but unfortunate) example of the oxygen paradox in action.

The potency of controlling redox balance is well illustrated in the context of cancer. On one hand, cancer cells increase reactive oxygen species and generate DNA damage, malignant transformation, and metabolic perturbations that provide the necessary resources for growth. On the other hand, cancer cells increase antioxidant activity during cancer progression to protect from excessive reactive oxygen species production, thus avoiding a toxic threshold. Disrupting this balance has been shown to have promising results in fighting certain breast and ovarian cancers, where chemotherapies involving high basal levels of reactive oxygen species have shown clinical promise. Exercise regimens also appear to initiate antioxidant mechanisms that may inhibit critical components essential to tumor growth. Nevertheless, these potential remedies must be applied cautiously because antioxidant supplementation has also been found to protect and encourage the grown of cancers. Identifying an individual’s redox status and applying oxidation/reduction-based therapies may 1 day prove to be a useful approach in cancer treatment.

The treatment of many disease states and age-related outcomes all have commonalities in restoring or maintaining redox balance. Diet-derived antioxidants show some promise in preventing certain types of cataracts, while the contributing to bone health and repair. Exercise, which stimulates cognitive and motor function, has been shown to affect metabolism and, in turn, affect neuroplasticity. The Oxygen Paradox is a story of imbalance in the redox state, while the French Paradox is a potential illustration of robust mechanisms that allow for the maintenance of redox balance. This review highlights the consequences of redox state imbalances that affect the ageing process, as well as many age-related diseases.
